# Joint attention in autism: A narrative review of assessment techniques from behavioral observation to artificial intelligence

**DOI:** 10.3758/s13428-026-02950-0

**Published:** 2026-03-02

**Authors:** Marwa Qaraqe, Elizabeth B Varghese, Inam Qadir, Dena Al-Thani, Chahnaz T Baroudi

**Affiliations:** 1 A-Sense Center of Excellence, College of Science and Engineering, Hamad Bin Khalifa University, Qatar Foundation, Doha, Qatar; 2 Renad Academy, Qatar Foundation, Doha, Qatar

**Keywords:** Autism spectrum disorder, Joint attention, Attention assessment, Social communication, Cognitive development

## Abstract

Joint attention (JA), the shared focus between two individuals on an object or event, plays a pivotal role in social communication, cognitive development, and language acquisition during early childhood. However, JA is frequently impaired in children with autism spectrum disorder (ASD), highlighting the need for precise assessment to support early diagnosis and intervention. This narrative review explores the evolution of JA assessment methods, tracing the shift from human-mediated techniques to technology-driven approaches, including artificial intelligence (AI). The study analyzes research indexed in major bibliographic databases between 2002 and 2024, categorizing findings into human-mediated and technology-assisted methods. Key aspects such as target populations, data collection processes, and validation strategies are examined. By highlighting the strengths and limitations of existing approaches, the review identifies future research directions that can advance JA assessment and inform early intervention strategies, ultimately benefiting children with ASD and their families.

## Introduction

Autism is a neurodevelopmental condition characterized by deficits in social communication and repetitive patterns of behavior. These characteristics are associated with differences in brain functionality and sensory processing, which influence how individuals perceive and interact with their environment (Faja & Dawson, 2017). As a spectrum, autism spectrum disorder (ASD) comprises a range of symptoms and responses, with each individual displaying unique reactions to people, objects, and situations. Over the years, its prevalence has increased globally.

A study conducted by Gerash University of Medical Sciences reveals that ASD’s worldwide prevalence was 0.6% (95% confidence interval (CI): 0.4–1). Subgroup analyses showed that ASD prevalence in America, Africa, Asia, Europe, and Australia was 1% (95% CI: 0.8–1.1), 1% (95% CI: 0.3–3), 0.4% (95% CI: 0.1–1), 0.5% (95% CI: 0.2–1), and 1.7% (95% CI: 0.5–6), respectively. This widespread prevalence highlights the critical importance of advancing precise and effective assessment methods for ASD. In particular, improved assessment of key developmental markers such as joint attention (JA) is essential for better understanding and supporting affected individuals and their families (Salari et al., 2022)

**Table 1:** Summary of reviews on JA assessment

Reference	Human-mediated	Technology-assisted	Area of focus	Limitations
Bruinsma et al. (2004)	$\checkmark$	x	IJA and its relation to language acquisition	Limited focus on technology-driven methods for JA assessment.
Korhonen et al. (2014)	$\checkmark$	x	JA assessment methods	Lack of empirical evidence for cross-validation of methods discussed.
Bottema-Beutel (2016)	$\checkmark$	x	JA assessment and its relation to language	Limited generalizability beyond language development context.
Mundy (2018)	x	$\checkmark$	Relation between JA and social cognition using imaging techniques	Narrow focus on imaging techniques without broader behavioral assessments.
Franchini et al. (2019)	$\checkmark$	x	IJA approaches	Limited attention to automated or technology-based approaches.
Chevalier et al. (2020)	x	$\checkmark$	JA assessment and intervention using social robots	Lack of diverse technology applications beyond social robots.
Bradley et al. (2023)	$\checkmark$	$\checkmark$	JA assessment in infants using human-mediated, eye-tracking, and EEG approaches	Lacks in-depth analysis of approaches leveraging VR, robotics, and computer vision

IJA: initiating JA, EEG: electroencephalogram, VR: virtual reality

According to the American Psychiatric Association (APA), a delay in or lack of attentional behaviors is one of the early indicators of ASD, highlighting the crucial role of attention in this context (Roehr, 2013). Attention, the cognitive process that allows individuals to selectively focus on specific aspects of their environment while ignoring others, is essential for prioritizing information and effectively allocating cognitive resources (Varghese et al., 2024). Sohlberg and Mateer (1987) classify attention into five primary categories based on the cognitive engagement required. They are focused attention (responding to stimuli by orienting attention), sustained attention (maintaining a continuous focus on a stimulus), selective attention (attending to a specific stimulus amidst distractions), shifting attention (transitioning attention between tasks of the same cognitive level), and divided attention (simultaneously responding to tasks with varying cognitive demands). Additionally, other forms of attention identified through empirical research include social attention, which involves directing attention toward social stimuli such as eye contact, and JA (Moore et al., 2014), which involves shared attention between individuals using verbal or non-verbal cues.

Among the various forms of attention, JA is particularly crucial as it is one of the earliest traits used to distinguish between children with ASD and typically developing (TD) children (Moore et al., 2014). JA involves looking back and forth between an object or event and another person, thereby connecting with that person (Mundy et al., 2003). This skill is fundamental for the development of social and communication abilities. Children with ASD typically exhibit delayed or absent social communication skills at each developmental stage. For instance, by 12 months, most children can look immediately in the direction that an adult points to, then look back at the adult and often mimic their expression (Zwaigenbaum et al., 2005). However, children on the autism spectrum may seem to ignore the adult. Thus, difficulties with JA are commonly observed in individuals with ASD, significantly affecting their capacity to engage in social interactions and develop social relationships effectively (Jones & Carr, 2004).

Children with ASD are routinely assessed in regard to JA during diagnosis because impairments in these skills are strongly associated with the condition (Moore et al., 2014). Although there is no standardized schedule for assessing JA, clinicians can use various methods based on the child’s age and developmental stage. JA typically develops between 9 and 12 months of age, beginning with infants responding to others’ cues and progressing to more active engagement, such as pointing and sharing the focus on objects (Mundy, 2018). This skill is crucial for language development, social interaction, and cognitive growth, as it helps children learn from their environment and connect with others. Hence, clinicians assess JA through observation and structured assessments, monitoring its progression to identify any delays. The number and choice of methods and instruments used should be tailored for the particular child to gain an accurate and complete picture of the JA abilities (Mundy et al., 2003).

Furthermore, JA plays a pivotal role in the educational context, underpinning several crucial developmental domains vital for effective learning. Proficiency in JA fosters language acquisition, enabling children to learn new words and concepts by sharing a common attentional focus with their teachers and peers (Wong & Kasari, 2012). JA is inextricably linked to cognitive growth, as it provides a rich context for learning, promoting the development of essential cognitive abilities such as problem-solving, reasoning, and executive functioning (Chiu et al., 2024). Hence, continuous assessment of JA within educational settings, using appropriate techniques, is crucial for understanding typical and atypical development. Such assessment also enables the early identification of subtle symptoms that may predispose individuals to later emerging developmental or learning issues, thereby supporting timely and targeted interventions throughout their academic trajectory (Bradley et al., 2023).

### Related reviews

A handful of reviews focusing on JA assessment are available in the literature; however, they primarily focus on human-mediated approaches. Table 1 summarizes the existing reviews. These existing reviews have made valuable contributions to synthesizing knowledge on JA assessment.

Bruinsma et al. (2004) provided an overview of JA behaviors in children with autism, examining empirical studies on eye gaze alternation, protodeclaratives, and proto imperatives. Their review offered insights into JA as a predictor of language acquisition. However, it was limited to human-mediated behavioral measures and did not address technological approaches. On the other hand, Korhonen et al. (2014) reviewed JA research from 2002–2013, examining the generality of JA impairment in autism and assessment methodologies. They found evidence of both impaired and intact JA skills, highlighting the variability within the autism population. However, they noted a largely homogeneous assessment methodology across studies, with none utilizing children’s individual interests in creating assessment situations. This highlights a limitation in capturing the full capabilities of individuals with autism.

To provide a more quantitative perspective, Bottema-Beutel (2016) conducted a meta-analysis and meta-regression to examine associations between JA and language in ASD and TD. Key strengths of this review include its quantitative synthesis of a large body of literature, examination of potential moderators of the JA–language relationship, and comparison of findings between ASD and TD. Limitations include the inability to examine all types of JA due to insufficient effect sizes for some categories, and the focus solely on associations with language outcomes rather than other developmental domains.

Moreover, Franchini et al. (2019) conducted a review focused specifically on the initiating JA (IJA) and related visual attention processes in infants with ASD. Their review synthesized findings from both retrospective and prospective studies, providing a developmental perspective on JA deficits in the first two years of life. A key strength was their examination of precursors to JA, such as dyadic engagement. However, the review was limited to studies published before 2018 and focused primarily on behavioral measures. Expanding the scope beyond behavioral assessments, Mundy (2018) review made significant contributions by examining the neurodevelopment of JA and its relevance to ASD. This paper provided valuable insights into the neural systems involved in JA from infancy through adulthood. However, its focus was primarily on neuroimaging studies rather than behavioral assessment methods.

As technology continues to advance, new methods for assessing JA have emerged. Chevalier et al. (2020) provided a novel perspective by reviewing methods to investigate JA using humanoid robots. They highlighted the benefits of using robots as dynamic "social stimuli" in naturalistic interactive scenarios, arguing that this approach provides more ecological validity than classical screen-based protocols while maintaining excellent experimental control. A limitation of this review is that it focused primarily on the potential of robot-assisted methods rather than providing an overview of existing JA assessment techniques.

Most recently, Bradley et al. (2023) conducted a scoping review to identify qualitative and quantitative measures of JA development in the first year of life. They categorized methods into qualitative (e.g., behavioral coding, standardized measures like the Early Social Communication Scale (ESCS)) and quantitative (e.g., eye-tracking, electroencephalogram(EEG)) approaches. A key contribution was the synthesis of information on how different methods can be combined to provide more comprehensive assessments. However, their study was limited to infant populations within the first year of life, overlooking critical developmental changes that occur later and excluding technology-based methods beyond eye-tracking and EEG.

**Fig. 1:**
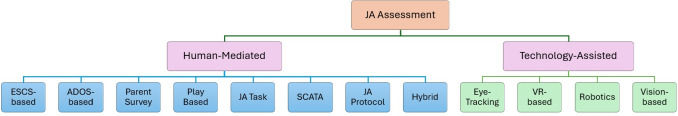
Taxonomy of JA assessment methods

To the best of our knowledge, none of the existing surveys provide a detailed discussion on JA assessment that comprises both human-mediated and technology-assisted perspectives, except for (Bradley et al., 2023). However, that study lacks an in-depth analysis of approaches leveraging virtual reality (VR), robotics, and computer vision. Additionally, it does not thoroughly explore how integrating these advanced methodologies could contribute to developing more robust and objective assessment techniques.

### Scope and relevance of the review

This review provides a narrative synthesis of the methodologies, technologies, and measures used in JA assessment across different target groups. It offers methodological clarity by identifying and categorizing human-mediated and technology-based approaches, highlighting their relative merits and limitations.

Emphasizing the use of advanced technologies such as computer vision, machine learning (ML), and robotics bridges gaps between developmental psychology and computational fields, fostering interdisciplinary integration. Additionally, the review guides future research by identifying open issues and proposing directions to address challenges like scalability, real-world applicability, and objective measures. It also ensures focus group representation by providing a nuanced understanding of target populations, making JA assessment approaches more inclusive and contextually appropriate across diverse cultural, age, and developmental profiles. Furthermore, the insights from this review have the potential to influence policy-making and clinical practice by advocating for accessible, effective, and evidence-based assessment methods.

## Methodology

### Research questions (RQ)

The methodology of this review is structured around addressing a set of carefully framed research questions that guide the analysis and synthesis of the existing literature. These research questions are as follows:

- RQ1- What are the methodologies used in JA assessment in the primary studies?
  - SRQ1- What are the human-mediated approaches for JA assessment?
  - SRQ2- Which technologies have been utilized for JA assessment in the primary studies?
- RQ2- Which groups are targeted in the primary studies?
- RQ3- What are the different measures utilized in the primary studies for JA assessment and what are the outcomes derived?
- RQ4- What are the open research issues and future directions inferred from the study?

These questions serve as a foundation to systematically explore the methodologies, target populations, assessment measures, and emerging challenges in the field of JA assessment. Based on these, the review has sought to create a wide-based understanding of the current landscape, identify gaps in the literature, and provide actionable recommendations for future research and development.

### Search strategy and study selection

To ensure a comprehensive synthesis of existing literature, data collection for this review was conducted systematically between January and December 2024. The scholarly articles included were sourced exclusively from peer-reviewed journals and high-impact conference proceedings to ensure the reliability and quality of the studies. Google Scholar, IEEE Xplore, Springer, and Web of Science served as the primary databases for identifying relevant research, using specific search queries tailored to the topic of JA assessment. These queries included terms such as ("Autis*" OR "Autism spectrum disorder" OR "ASD") AND ("Joint attention assessment" OR "Measuring joint attention" OR "Eye-tracking for joint attention" OR "virtual reality for joint attention assessment" OR "Robotic systems for joint attention assessment" OR "Computer vision-based joint attention assessment"). In addition, cross-referencing was employed to identify highly cited articles within the initial results, ensuring the inclusion of recent and significant contributions to the field.

#### Inclusion and exclusion criteria

The review adopted clear inclusion criteria to maintain the relevance and rigor of the studies analyzed. Only articles published between 2002 and 2024 were included, focusing specifically on JA assessment in the context of ASD. These studies were required to use validated and rigorously evaluated tools and methodologies. Exclusion criteria were equally stringent, with mini-reviews, unvetted articles, and commentaries excluded due to their lack of original data or detailed analysis. In addition, studies that focused solely on JA interventions without assessment were excluded. As a result, a total of 71 primary studies were identified for the review.

**Fig. 2:**
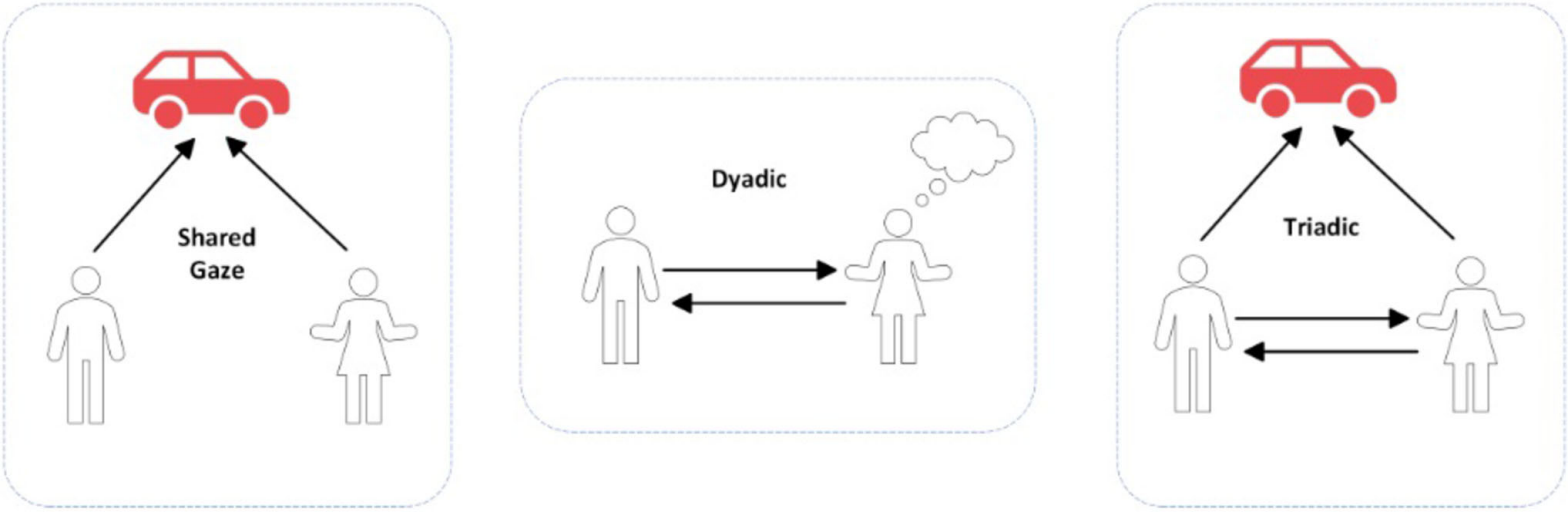
Schematic representation of the levels of JA

### Organization and taxonomy

The taxonomy employed in this review organizes JA assessment methods into two primary groups: human-mediated and technology-assisted approaches. Human-mediated approaches to JA assessment rely on direct interaction between individuals, typically involving caregivers, clinicians, or researchers who observe, prompt, and evaluate JA behaviors (Mundy et al., 2003; Lord et al., 1989). On the other hand, technology-assisted approaches utilize digital tools, sensors, or artificial intelligence (AI) to assess JA objectively (Ko et al., 2023). This categorization is visually illustrated in Fig. 1, providing a clear framework for analyzing the strengths, limitations, and applicability of each method. By adhering to this methodological framework, the review ensures a thorough and unbiased evaluation of existing literature, offering valuable insights and a foundation for future research in JA assessment.

## Joint attention: A conceptual overview

Before language development, infants acquire various communication skills. A critical aspect of this process is learning to jointly attend to the same object/event with another individual during social interactions. The capacity for purposeful social attention coordination, known as JA, gradually develops from the early months of the first year of life through childhood. Similar to language development, the behaviors of JA in infancy manifest in both receptive and expressive forms (Mundy et al., 2009).

The receptive behavior of JA is responding to JA (RJA), where infants follow the direction of another’s gestures and gaze to share a common reference point. Fully developed RJA enables toddlers and infants to use the gaze of others to identify the meaning of actions, such as language, intentions, or emotions (Dawson et al., 2005). In contrast, IJA is the expressive behavior, which enables infants to use eye contact and gestures to direct the attention of others to events, objects, or themselves. This form functions to nonverbally communicate and share a child’s experiences or interests with others (Mundy et al., 2003). Both RJA and IJA are behaviorally well developed by 9 months and continue to mature into the second year of life. While the onset of these abilities is less well understood, evidence suggests that RJA may begin as early as 2–4 months of age (Mundy, 2018).

JA is essential for understanding autism due to its role in the social attention model, which posits that autism is characterized by atypical attention to people in early life. This reduced social attention results in an early social learning disability, impacting the development of social communication and cognitive skills in children with autism (Dawson et al., 2005; Mundy et al., 1984). Consequently, impairments in social attention are considered a core symptom of autism (Dawson et al., 2005; Mundy et al., 2009). This is manifested as impaired JA, where there is a diminished ability to effectively coordinate visual, auditory, or mental attention with a social partner (Mundy et al., 2009). Therefore, assessment of JA abilities is essential, as it helps to inform appropriate intervention strategies for individuals with ASD. Clinicians and researchers have devised various approaches to measure JA skills, which are discussed in this review. These assessment methods primarily focused on evaluating two key JA behaviors: IJA and RJA.

**Table 2:** Human-mediated approaches

Author	Participants	Methodology	Measures	Remarks
Wetherby and Prizant (2002)	2000 children	Parental survey	CSBS-DP	Aims to improve early identification of communication delays before age 2 years.
Charman (2003)	18 ASD children	JA task	Gaze switching, Goal direction	Greater proficiency in gaze switching, less severe social and communication symptoms at 42 months. JA behaviors are associated with later language and symptom severity.
MacDonald et al. (2006)	26 ASD and 21 TD (total of 47 children)	JA protocol	IJA, RJA	Children with ASD had relatively minor deficits in RJA compared to TD children, but more severe deficits in IJA, with over half the children with autism having low or no JA initiations.
Drew et al. (2007)	Sample 1- 17 ASD, Sample 2- 29 ASD	SCATA	Form, Function, Role, Complexity	Non-verbal social communication behavior assessment.
Kidwell and Zimmerman (2007)	36 children	Play-based	Show actions	JA is a practical, social accomplishment that children navigate with a sophisticated grasp of social contingencies.
Sullivan et al. (2007)	51 ASD	Hybrid	RJA	Defects in RJA in 14 months diagnosed with ASD later. Poor RJA led to later language impairment.
Presmanes et al. (2007)	81 children (46 younger siblings of ASD children, 35 younger siblings of TD children)	Play-based	RJA	Younger siblings of ASD have lower RJA scores than younger siblings of TD.
Roos et al. (2008)	20 ASD	Hybrid	IJA, RJA	Empirical support for naturalistic play for JA assessment. Behaviors elicited during ESCS are similar to behaviors during naturalistic play.
Chiang et al. (2008)	28 ASD, 24 DD, and 52 TD (total of 104 children)	ESCS	IJA, RJA, ISI, RSI	Children with autism displayed deficits in JA ability, particularly high-level skills. Exhibited a marked deficit in communication frequency compared to TD infants.
Paparella et al. (2011)	35 ASD, 18 TD	ESCS	IJA, RJA	Notable differences in the acquisition sequence of JA skills between children with ASD and TD children.
Schietecatte et al. (2012)	23 ASD	ESCS	IJA, RJA	RJA correlated with intention understanding, frequency of IJA linked to attention disengagement, and, to a slightly lesser extent, social preference.
Maljaars et al. (2012)	36 children with ASD and ID, 26 with ID and 34 TD (total of 96 children)	ADOS 1 and 2	IJA, RJA	Notable distinctions in language skills across the three groups, with JA showing a robust correlation with concurrent language proficiency in low-functioning children with ASD.
Bean and Eigsti (2012)	18 ASD and 24 TD	Play-based	RJA	JA scores correlated with language and theory of mind measures in ASD group.
Wong and Kasari (2012)	27 ASD, 28 other DD preschoolers	Hybrid	IJA, RJA, Engagement	Children with autism spent more time unengaged and exhibited lower levels of symbolic play and JA compared to their peers.
Elison et al. (2013)	51 TD infants	Play-based	RJA	9 to 10 months represents the time of maximal individual differences in RJA.
Harrison et al. (2016)	1061 ASD	ADOS 1 and 2	Spontaneous IJA, RJA	VIQ is fully mediated in the relationship between JA and adaptive functioning, while partially mediated by NVIQ.
Mundy et al. (2017)	86 school-aged children	Parental Survey	C-JARS	Evaluate JA skills in children, taking into account verbal behavior.
Panganiban (2017)	28 ASD	Hybrid	IJA, IBR	Structured and unstructured play effective in measuring IJA. Stronger correlation between IJA and IBR.
Nowell et al. (2018)	Sample 1 81 TD, 57 DD, 122 ASD. Sample 2 – 19 ASD	JA Protocol	IJA, RJA	Demonstrated good psychometric properties for clinical utility. Correlated with ADOS items measuring JA skills.
Adamson et al. (2019)	40 TD, 58 ASD, and 46 with DD (total of 144 toddlers)	ESCS		Weaker JA abilities, joint engagement during parent-toddler interactions, and expressive language skills for toddlers later diagnosed with ASD.
Cilia et al. (2020)	50 ASD and 50 TD preschoolers	ESCP (French adaptation of ESCS)	IJA, RJA	ASD children are less responsive than TD children, Multiple deictic cues affect ASD children’s response behaviors, No difference in the number of IJAs between groups.
Gabouer and Bortfeld (2021)	Deaf and hearing children, Number not specified	Play-based	IJA, RJA, Verification of JA	New Coding Protocol, Software for JA coding.
Sano et al. (2021)	153 ASD	ADOS 2	IJA, RJA	Significant association between severe JA deficits and low intelligence, with greater severity of JA deficits corresponding to lower scores in problem-solving ability and knowledge of things.
Montagut-Asunción et al. (2022)	32 ASD infants	ESCS	IJA, RJA, IBR, RBR	Higher IJA at 8 months and RJA at 12 months correlated with an increased risk of ASD at 18 months.
Thurman and Dimachkie Nunnally (2022)	30 ASD, 21 FXS	Play-based	IJA, RJA	JA strongly correlated with all language measures in boys with autism. JA performance was negatively associated with autism symptomatology.
Lasch et al. (2023)	210 TD	Play-based	RJA	DJAA scores were associated with later verbal abilities and parent-reported social responsiveness. Associations between RJA and executive function.
Dakopolos et al. (2024)	42 ASD	Hybrid	IJA, RJA	Children’s JA related to mother behavior and contextual factors.
Moerman et al. (2024)	49 preterm ASD, 48 siblings of ASD	Hybrid	IJA, RJA	Early play behavior resulted in later expressive language, which was absent in preterms.

$^{1}$CSBS-DP: Communication and Symbolic Behavior Scales Developmental Profile, ADOS: Autism Diagnostic Observation Schedule, DD: Developmental Delays, ID: Intellectual Disabilities, ESCS: Early Social Communication Scales, SCATA: Social Communication Assessment for Toddlers with Autism, C-JARS: Childhood JA Rating Scale, IBR: Initiating Behavioral Request, ISI: Initiating Social Interaction, NVIQ: Non-Verbal Intelligence Quotient, RBR: Responding to Behavioral Request, RSI: Responding to Social Interaction, VIQ: Verbal Intelligence Quotient, DJAA: Dimensional JA Assessment

When assessing JA, three distinct levels are usually considered, each representing a progressively more sophisticated understanding of the shared attentional experience (Bruinsma et al., 2004). The most basic level is shared gaze, in which two individuals simultaneously look at the same object, without necessarily realizing that their attention is shared. The next level is dyadic attention, which involves a conversational exchange of facial expressions, vocalizations, and speech (in the case of adults). This typically emerges around two months of age in infants (de Souza et al., 2022). The highest level is triadic attention, where two individuals not only attend to the same object but also understand that their attention is coordinated. This requires an understanding of gaze direction and intentionality, which is marked by the individual looking back and forth between the object and the social partner (Meindl & Cannella-Malone, 2011). Figure 2 portrays a schematic representation of the three levels.

The frequency of employing JA assessments in clinics and schools varies based on several factors, including the child’s age, developmental stage, and individual needs. In clinical settings, the assessments might be conducted during initial evaluations of a child’s developmental milestones and social communication skills, follow-up visits, or as part of ongoing therapy. For children with developmental concerns or suspected ASD, assessments could be more frequent, potentially every few months or as needed based on progress and intervention plans (Roehr, 2013). On the other hand, in educational settings, JA assessments are usually integrated into broader developmental evaluations, special education assessments, or periodic reviews. The Individuals with Disabilities Education Act (IDEA) mandates that special education assessments, including those for JA, occur at least annually during Individualized Education Program (IEP) reviews or when specific concerns arise (Davis et al., 2005). Thus, by understanding the levels, significance, specific behaviors, and various approaches to assessing JA, researchers and practitioners can better evaluate and address JA deficits in children with ASD, thereby improving social communication, language development, and overall social functioning.

## Results

This section presents a narrative review of the evolution of JA assessment strategies, transitioning from human-mediated methods to technology-assisted approaches. The results are systematically categorized based on the research questions outlined in Section II-A. This review is based on the 71 primary studies identified, of which 28 are categorized as human-mediated, and the remaining 43 constitute technology-assisted approaches. A summary of human-mediated approaches is provided in Table 2, highlighting their methodologies and applications, while technology-assisted approaches, comprising advancements such as eye-tracking, VR, robotic systems, and computer vision, are summarized in Table 3.

**Table 3:** Technology-assisted approaches

Author	Participants	Technology Used	Measures	Remarks
Navab et al. (2012)	52 ASD infants	Eye-tracking	RJA	Eye-tracking measures were positively correlated with RJA during the distal ESCS task, establishing the construct validity of the eye-tracking assessment.
Chawarska et al. (2012)	54 ASD, 22 DD, 48 TD toddlers	Eye-tracking	RJA	ASD showed decreased attention compared to DD and TD, and related to lower nonverbal functioning and symptom severity.
Chawarska et al. (2013)	117 ASD infants	Eye-tracking	RJA	6-month-old infants later diagnosed with ASD exhibited less fixation duration towards the social scene and face.
Falck-Ytter et al. (2012)	Study 1- 13 ASD 9 children with other developmental problems, and 14 TD children Study 2- Participants of study 1 + 15 TD toddlers	Eye-tracking	RJA	Duration of the first fixations at the objects in the scene indicated a notably weaker initial processing bias for attended objects in children with ASD compared to children with TD and non-autistic children with DD.
Pfeifer-Lessmann et al. (2012)	20 TD adults	VR	IJA	Compare JA capabilities during human–human and human–avatar interactions.
Swanson and Siller (2013)	21 ASD, 24 TD children	Eye-tracking	RJA	ASD showed difficulties in gaze following compared to their TD counterparts.
Riby et al. (2013)	78 child and adult participants(26 ASD, 15 with Williams syndrome, and 37 TD)	Eye-tracking	RJA	ASD had longer fixation duration compared to TD individuals during gaze-following tasks.
Gillespie-Lynch et al. (2013)	24 ASD and 42 TD children	Eye-tracking	RJA	Participants with ASD demonstrated diminished gaze-following abilities compared to TD children.
Guo and Feng (2013)	92 TD children	Eye-tracking	RJA	Significant improvements in JA and children’s word learning in intervention groups compared to the baseline group.
Falck-Ytter et al. (2015)	Study 1- 13 ASD, 9 DD, and 14 TD children. Study 2- 15 TD toddlers.	Eye-tracking	RJA	In intellectually low-functioning ASD, gaze following is intact with a weaker duration of first fixations compared to DD and TD.
Thorup et al. (2016)	64 ASD infants	Eye-tracking	RJA	Aimed to determine if objects perceived as highly interesting by children with ASD would influence gaze-following behaviors.
Congiu et al. (2016)	25 ASD, 25 TD children	Eye-tracking	RJA	ASD children are less accurate in gaze-following and spend less time looking at the symbolic object
Billeci et al. (2016)	17 ASD and 15 TD toddlers	Eye-tracking	IJA, RJA	No differences between toddlers with ASD and TD toddlers in the RJA task, but significant differences in the IJA tasks.
Little et al. (2016)	31 ASD and 33 TD young adults	VR	IJA, RJA	Similar target recognition between IJA and RJA conditions for both groups, but the ASD group exhibited poorer overall recognition memory.
Mundy et al. (2016)	32 ASD, 27 ADHD, and 23 TD	VR	IJA, RJA	TD and ADHD groups showed better recognition memory for pictures studied in the IJA condition compared to the RJA condition, a pattern absent in the ASD group.
Franchini et al. (2017)	25 ASD and 21 TD preschoolers	Eye-tracking	RJA	Decrease in overall time spent on communicative cues and reduced RJA in the ASD group compared to their TD counterparts.
Thorup et al. (2017)	64 ASD infants	Eye-tracking	RJA	Infants in the high-risk group were more likely to follow gaze by turning head compared to gaze shifting
Nyström et al. (2017)	61 ASD and 18 TD infants	Eye-tracking	RJA	Children in the high-risk group exhibited less fixation on the adult during the time window of 300–1000 ms after the adult initiated direct gaze.
Parsons et al. (2017)	124 ASD infants	Eye-tracking	RJA	Infants who later developed ASD followed gaze as frequently as their TD peers, but spent less time engaged with either object. Spending more time on faces and less on objects linked to lower concurrent or subsequent verbal abilities.
Caruana et al. (2017)	16 TD adults	VR	IJA, RJA	Intention monitoring during communicative gaze shifts influenced subsequent JA behavior.
Vivanti et al. (2017)	77 preschoolers (35 ASD, 22 with Williams syndrome, and 20 TD)	Eye-tracking	RJA	ASD spent less time looking at the actor’s face and exhibited reduced gaze following when compared to TD participants.
Billeci et al. (2017)	14 ASD children	Eye-tracking	IJA, RJA	Multimodal approach to characterize JA-related brain circuitries and visual patterns in ASD children with ASD, and to monitor longitudinal changes in response to interventions.
Caruana et al. (2018)	17 ASD and 17 TD adults	Eye-tracking	RJA	Difficulties in gaze following were observed in ASD adults compared to TD.
Amaral et al. (2018)	15 ASD adults	VR	RJA	Measure the frequency of accurate JA for intervention in subsequent sessions.
Kumazaki et al. (2018)	30 ASD and 38 TD children	Robotic systems	RJA	Robotic intervention is found to be better than human intervention in children with ASD
David et al. (2018)	5 ASD children	Robotic systems	RJA	The performance increased by increasing the JA cues in child–robot interaction
Zhang et al. (2018)	8 TD adults	Vision-based	RJA	RJA patterns were detected using a vision-based system with RGB and Kinect cameras.
Cilia et al. (2019)	28 ASD, 28 with developmental communication disorders, and 28 TD children	Eye-tracking	RJA	Static stimuli facilitated the precise identification of AOIs and revealed similarities in visual exploration patterns between children with ASD and TD. Differences in visual exploration during dynamic stimuli suggested that the nature of the stimulus may influence JA behaviors differently across groups
Jyoti and Lahiri (2019)	20 ASD and 20 TD children	VR	RJA	Identify JA deficits in children with ASD compared to TD children.
Ravindran et al. (2019)	12 ASD children	VR	IJA, RJA	Positive skill improvements in the total number of interactions, initiation of interactions, and use of eye contact
Mehmood et al. (2019)	8 ASD children	Robotic systems	IJA, Imitation	IJA of children with ASD in the vision space is from right to left.
Griffin and Scherf (2020)	40 ASD and 49 TD adolescents	Eye-tracking	RJA	ASD adolescents showed difficulties in gaze following compared to their TD peers.
Zheng et al. (2020)	23 ASD children	Robotic systems	RJA, STAT	Emphasized the need for individualized intervention for autistic children.
Ambrose et al. (2021)	1 ASD child	Eye-tracking	IJA	Differences in gaze use and interactive JA behaviors between the therapists working with the child and within the child’s dyadic interactions
Amat et al. (2021)	9 ASD and 9 TD children	VR	RJA	Effective in identifying JA skill deficits in children with ASD compared to their TD peers.
Zhang et al. (2022)	143 ASD and 113 TD children, 43 TD adults	Eye-tracking	RJA	Automated tool to quantify RJA.
de Belen et al. (2023)	77 ASD children	Eye-tracking	RJA	Higher accuracy in gaze following and better early cognition and adaptive behaviors in children with ASD, while less accurate gaze following was associated with more severe ASD symptomatology.
Temeltürk et al. (2023)	30 ADHD and 30 TD children	Eye-tracking	RJA	Children with ADHD had higher autistic traits and lower PoF on the object AOI, indicating RJA deficits compared to TD children.
Yazdanian et al. (n.d.)	12 ASD children	VR	IJA, RJA	Virtual cross-validation tool for C-JARS assessment method.
Prakash et al. (2023)	No details	Vision-based	IJA, RJA	DL models were proposed to detect the JA assessment characteristics.
Ko et al. (2023)	45 ASD and 50 TD children	Vision-based	IJA, RJA	DL models for ASD symptom severity detection using JA assessment method.
Ambarchi et al. (2024)	56 ASD and 34 TD children	Eye-tracking	IJA, RJA	Children with ASD displayed reduced attention to socially salient stimuli compared to TD children across SBR videos and specifically during JA bids.
Ozdemir et al. (2024)	56 ASD and 56 TD toddlers	Eye-tracking	RJA	Visual attention towards JA AOIs connected to language and cognitive development.

$^{2}$ADHD: Attention Deficit Hyperactivity Disorder, STAT: Screening Tool for Autism in Toddlers and Young Children, AOI: Area of Interest, SBR: Shared Book Reading

### RQ1- JA assessment methodologies

The detailed analysis of methodologies broadly classified as human-mediated and technology-assisted is presented in this section.

#### SRQ1-Human-mediated approaches

This section aims to uncover the methods and techniques that rely on non-technological (psychologically based) means to evaluate an individual’s ability to engage in JA behaviors. These methods typically involve direct observation of the individual’s behavior in structured or unstructured settings, often using standardized tasks or tasks designed by the assessor (Charman, 2003). These assessments are usually administered in person by trained professionals, such as psychologists, speech therapists, or educators, and may involve interaction with the individual being assessed and/or their caregivers (Charman, 2003; Mundy et al., 2003). In most cases, the assessment sessions are video recorded, allowing the assessor to review the footage post-assessment to analyze behaviors and generate an evaluation score.

**Fig. 3:**
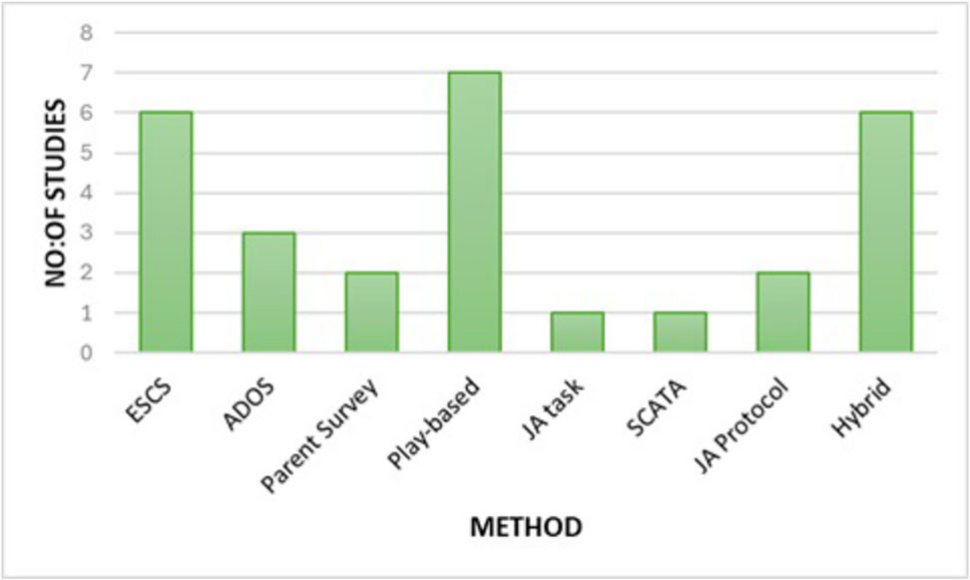
Category of studies in human-mediated approaches

This review identifies 28 primary studies in the category of human-mediated assessment, which were further classified into sub-categories as illustrated in Fig. 3. As evident from the figure, a significant proportion of the methods are based on Play-based. The following subsections provide a detailed overview of the studies that utilized the identified approaches, highlighting their methodologies.

- *ESCS*: One of the most common and clinical JA assessment methods is the ESCS developed by Mundy et al. (2003). The ESCS is a structured observation measure designed for children aged 8 to 30 months. It assesses individual differences in nonverbal communication skills and can be used with TD children or those with developmental delays (DD) within this age range. Originally developed based on Piagetian cognitive stages and pragmatic-functional orientation (Mundy et al., 1984), the ESCS comprises 25 structured situations to encourage interaction between an adult tester and the child. Behaviors are coded and summarized based on developmental stage, communicative goal, and initiation or response to interaction bids, resulting in a social-communicative profile of the child’s highest levels across various communicative functions. The studies (Adamson et al., 2019; Chiang et al., 2008; Montagut-Asunción et al., 2022; Paparella et al., 2011; Schietecatte et al., 2012), and (Cilia et al., 2020) employed the ESCS to evaluate JA and investigate its correlation with essential social and cognitive abilities crucial for daily functioning, including social communication, language development, and intelligence. The study by Montagut-Asunción et al. (2022) assessed JA at two developmental stages (8 and 12 months) to investigate whether their JA skills were associated with early signs of ASD at 18 months. Another study by Paparella et al. (2011) used ESCS to analyze whether the timing and sequence of JA during IJA and requesting skill acquisition during RJA in children with ASD differ from those in TD children. Cilia et al. (2020) employed the French adaptation of the ESCS protocol, known as ESCP (Guidetti & Tourrette, 1993), to investigate RJA and IJA in preschoolers diagnosed with ASD in comparison to TD children.
- *ADOS:* Due to the prevalent impairment of JA among children with ASD, assessments of JA, including both IJA and RJA, have been integrated into ASD-specific diagnostic tools such as the ADOS (Lord et al., 1989). Leveraging information on JA from widely used measures like the ADOS allows researchers to measure JA in large ASD samples with relatively minimal resources, thereby facilitating research across genetic and epidemiological (Harrison et al., 2016). Studies employing factor analysis on ADOS subfactors have identified an additional JA factor, comprising various social behaviors beyond JA, such as behavioral requesting (e.g., pointing, gesturing) and overall social functioning (Gotham et al., 2007, 2008; Oosterling et al., 2010; Robertson et al., 1999). The study proposed by Harrison et al. (2016) investigates the cognitive and adaptive correlates of an ADOS-derived JA composite in children diagnosed with ASD. The research by Maljaars et al. (2012) utilized ADOS modules 1 or 2 for JA assessment and explored the association of JA with language development. Sano et al. (2021) utilized JA assessment via ADOS-2 to investigate its correlation with intelligence levels in children diagnosed with ASD.
- *Parental survey* The Communication and Symbolic Behavior Scales Developmental Profile (CSBS- DP) (Wetherby & Prizant, 2002) stands out as one of the widely employed parental survey measures. Here, a screening tool was designed to identify early communication delays in children ages 6–24 months. The checklist measures seven key language predictors identified by research: emotion/eye gaze, communication, gestures, sounds, words, understanding, and object use. Another parent-report measure is the Childhood JA Rating Scale (C-JARS) (Mundy et al., 2017), aimed at evaluating JA skills in children, considering verbal behavior. Initial findings suggest that symptoms associated with reduced JA and the spontaneous sharing of experiences with others can be evaluated using this measure in children and adolescents with ASD. The C-JARS is noted to assess a distinct aspect of the social characteristics of children with ASD. While these methods have shown effectiveness, they rely solely on parent reports, which may result in overestimating or underestimating abilities.
- *Play-based* Researchers have increasingly recognized the importance of examining JA skills in spontaneous, naturalistic forms (Abdelaziz et al., 2025; Bean & Eigsti, 2012; Deák et al., 2008; Elison et al., 2013; Gabouer & Bortfeld, 2021; Kidwell & Zimmerman, 2007; Lasch et al., 2023; Presmanes et al., 2007; Thurman & Dimachkie Nunnally, 2022). These assessments enable the observation of JA behaviors as they unfold dynamically within real-world social contexts, providing valuable insights into the practical manifestation of this pivotal ability. Kidwell and Zimmerman (2007) demonstrated that JA is not merely a psychological process but a fundamentally interactional one, intricately woven into ongoing social activity. The study elucidates the intricate practical procedures children employ to draw and sustain another’s attention through meticulous analysis of young children’s actions of showing objects to others. It highlights that JA extends beyond co-orienting to an object, encompassing the crucial act of conveying the intended social purpose of the interaction. The authors Bean and Eigsti (2012) developed and validated a naturalistic 6-prompt assessment of JA skills for school-age children and adolescents with ASD. The assessment consisted of six examiner-initiated prompts embedded throughout a testing session, designed to elicit RJA, including both verbal (e.g., calling the participant’s name) and nonverbal (e.g., offering a pen) cues, as well as prompts requiring single or dual attentional shifts. The examiner administered these prompts when the participant was visually engaged with another task or object, not when directly looking at the examiner. Responses were scored based on several criteria: engaging in triadic attention, looking at the examiner’s face, making eye contact, and offering a spontaneous, relevant verbalization. The naturalistic play setting of parent–child interactions was explored by Presmanes et al. (2007) and Gabouer and Bortfeld (2021) to objectively identify and characterize instances of JA. The RJA capacity of children was investigated by Presmanes et al. (2007) by giving different verbal and non-verbal cues. Gaze shifting, point, gaze block, and their combinations were used to determine the attention responses and scores. On the other hand, Gabouer and Bortfeld (2021) assessed JA in three steps: intention, response, and active verification, each with specific criteria and time windows. In the intention step, a bid to initiate JA is evaluated based on specific indicators like visual focus and gestures toward an object. The response step requires the non-initiating partner to reply within five seconds, with successful responses including gaze-shifting or engagement lasting at least three seconds; bids are marked as failed if there’s no response within this period. The active verification step involves the initiator confirming the target’s focus through gaze changes or vocal responses, demonstrating intentionality in the interaction. The scheme also considers instances that do not qualify as bids, particularly when verification fails, and allows for the analysis of multimodal cues used in the bids. The assessment by Thurman and Dimachkie Nunnally (2022) involved presenting the child with various toys and objects while the examiner performed specific gestures (giving, blocking, teasing, or pointing/gazing) to elicit JA responses. The child’s gaze behavior was observed following each gesture, with points awarded for demonstrating JA within a 4-s window. The study proposed by Elison et al. (2013) developed the Dimensional JA Assessment (DJAA), a naturalistic play-based assessment of RJA in infants. The assessment involved a series of hierarchically ordered prompts to elicit gaze shifts, head turns, and other cues, with responses scored on a 0–4 scale, allowing for a detailed evaluation of RJA performance. DJAA was also utilized in Lasch et al. (2023) to observe TD children, and the study revealed significant associations between DJAA scores and later verbal abilities and social responsiveness. A semi-structured parent–child play session was utilized by Abdelaziz et al. (2025) to examine the associations between JA, joint engagement, and language development. Each child participated in a 30-min play session comprising both free and structured play. The first 5 min and the last 10 min were designated for free play, allowing caregivers to interact naturally with their children. The middle 15 min followed a structured format, incorporating 12 play-based activities, including pretend play with dolls, interactive play with a ball or truck, imitative actions, and JA behaviors such as pointing and reaching. To maintain consistency, caregivers were provided with instructional cards from the experimenter, guiding them through the activities.
- *JA task* The JA task consists of two components: the activated toy task and the goal detection task (Charman, 2003). In the activated toy task, mechanical toys were used to elicit ambiguous responses from children, who were seated between their mother and the experimenter. The primary behavior assessed was the child’s ability to shift gaze between the toy and the adult, serving as an indicator of JA. The goal detection task comprised a blocking task and a teasing task, designed to further evaluate the child’s capacity for shared attention and interpretation of social cues. The study by Charman (2003) examined the importance of JA in infants with ASD at 20 months and its relationship with language development and symptom severity at 42 months. A total of 18 children with ASD participated, undergoing experimental assessments such as spontaneous play tasks and JA tasks. The findings demonstrated that children who exhibited more frequent gaze switching in the activated toy task at 20 months showed less severe social and communication symptoms at 42 months. Moreover, JA behaviors during the activated toy task were significantly associated with later language outcomes and symptom severity, emphasizing the crucial role of JA in the developmental trajectory of ASD.
- *SCATA* Social Communication Assessment for Toddlers with Autism (SCATA) is a semi-structured method for assessing JA, proposed by Drew et al. (2007). This approach is tailored to evaluate non-verbal communication, including atypical and early communication, in young toddlers with ASD. The assessment involves a combination of structured tasks like free play, turn-taking activities, and specific prompts, as well as unstructured elements such as the use of activated musical toys and bubbles. The SCATA aims to measure the frequency, form, and function of communication in toddlers with ASD, with scoring dimensions consisting of form, function, role, and complexity of communicative acts.
- *JA protocol* JA Protocol (Watson et al., 2003) is a live-coded behavioral measure designed to assess children’s ability to follow and initiate JA. The JA Protocol consists of 16 structured trials alternating between eight opportunities to observe RJA and eight opportunities to observe IJA. During administration, an examiner presents a series of engaging toys and activities while providing specific prompts to elicit JA behaviors from the child. These behaviors are then coded live by trained examiners using a dichotomous pass/fail scoring system for each trial. The Behavioral Assessment of JA protocol, which MacDonald et al. (2006) developed, evaluated JA responses and initiation in children with ASD and TD children aged 2–4 years. The assessment included subtests to measure RJA (following a point to pictures) and IJA (through gaze shifts, gestures, and verbalizations during toy activation and book presentation tasks). Nowell et al. (2018) conducted an extensive psychometric evaluation of this protocol, involving 260 children across various developmental profiles, including ASD, DD, and TD. The protocol involves administering a series of structured prompts to elicit JA behaviors, which are then coded live by trained examiners. Their findings demonstrated the protocol’s strong psychometric properties, including good reliability, validity, and a clear two-factor structure representing RJA and IJA.
- *Hybrid approaches* The studies identified as hybrid employed a combination of two or more methods discussed above for JA assessment. These hybrid approaches integrate multiple human-mediated strategies to leverage the strengths of individual methods, providing a broad evaluation of JA behaviors. A summary of the hybrid approaches, along with their constituent methods, is presented in Table 4, offering insights into the specific combinations used in the identified studies. The study by Sullivan et al. (2007) aimed to examine the RJA in toddlers at high risk for ASD using a prospective, longitudinal design. The researchers adapted the Butterworth task, CSBS-DP, and the ADOS to weave their assessment. Roos et al. (2008) embarked on a study that wove together the threads of standardized coding and naturalistic observation. They examined whether the JA behaviors exhibited during spontaneous play aligned with those unveiled through the structured lens of the ESCS. Notably, the ESCS elicited a richer tapestry of initiated JA episodes, while the play sample provided a window into the nuanced responses to JA bids. This convergence of evidence lent credence to the notion that naturalistic sampling could serve as a viable alternative or complement to standardized measures. The Structured Play Assessment (SPA), along with classroom observation and ESCS were employed by Wong and Kasari (2012), which provided a window into the manifestation of JA deficits in naturalistic educational settings. Observing children with autism and those with other DD in preschool classrooms, the study found that children with autism spent more time unengaged and exhibited lower levels of symbolic play and JA compared to their peers. Crucially, teachers rarely focused on teaching or responding to these pivotal skills, highlighting the need for classroom-based interventions that target JA and play abilities. SPA and ESCS, along with Unstructured Caregiver Child Interaction (MCX), were utilized by Panganiban (2017) to track specific JA skills such as coordinated looks, alternating gazes, points, gives, shows, and language use. ESCS, along with CSBS-DP, was used in a longitudinal study conducted by Moerman et al. (2024) to assess the predictive value of JA growth rate for language abilities and the likelihood of autism. The study focused on preterm children and siblings of children with autism. In contrast, social contexts such as teaching, competing demand, and free play were utilized in Dakopolos et al. (2024) to examine the relationship between JA and maternal attention. The study included both autistic children and their mothers. In the teaching task, mothers were instructed to help their children build a complete structure using building blocks. The competing demand task was designed to observe the child’s response to a potentially tempting object, such as an iPad, when the mother was occupied with other tasks. During free play, the mother and child engaged in naturalistic play with toys.

**Table 4:** Hybrid approaches-Summary of combination

Study	Methods combined
Sullivan et al. (2007)	Butterworth task, CSBS-DP, ADOS
Roos et al. (2008)	ESCS, Naturalistic Play
Wong and Kasari (2012)	Classroom observation, SPA, ESCS
Panganiban (2017)	ESCS, SPA, Unstructured Play
Dakopolos et al. (2024)	Demanding Tasks, Teaching Tasks, Free Play
Moerman et al. (2024)	ESCS, CSBS-DP

**Fig. 4:**
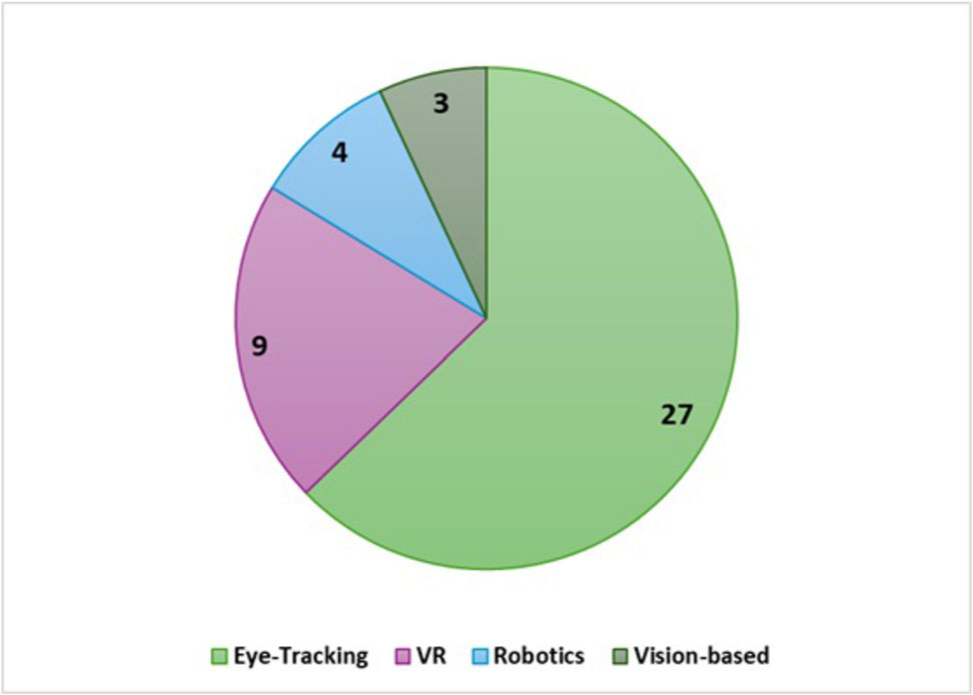
Categories and number of studies in technology-assisted approaches

#### SRQ2-Technology assisted approaches

Advancements in technology, particularly in computer graphics, have prompted researchers to transition from traditional methods to computer-based technological approaches for assessing JA (Jyoti et al., 2020). Furthermore, the progress in human-computer interaction has facilitated enhanced communication between humans and computers, leading to the development of technologies such as eye-tracking, face recognition, VR, and robotics (Zhang et al., 2018). As a result, recent research has predominantly focused on integrating these technologies for the task of JA assessment. This section delves into the various technology-based approaches for JA assessment, including eye-tracking, VR, robotic systems, and computer vision. A total of 43 primary studies were identified in this category, with 27 focusing on eye-tracking technology. Figure 4, presents the subcategories and their cardinalities.

**Fig. 5:**
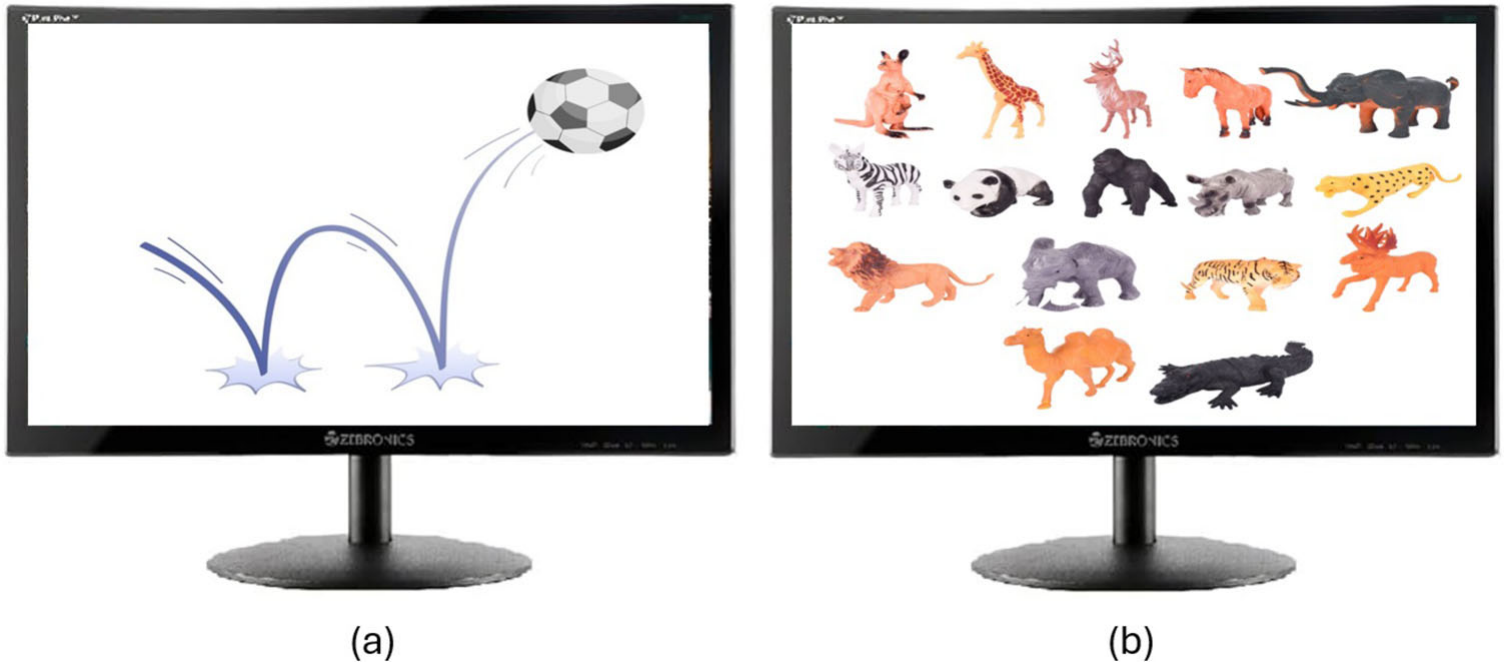
(**a**) A sample video clip representing dynamic stimuli showing the bounce of a ball. (**b**) A sample image with animal pictures depicting static stimuli

**Fig. 6:**
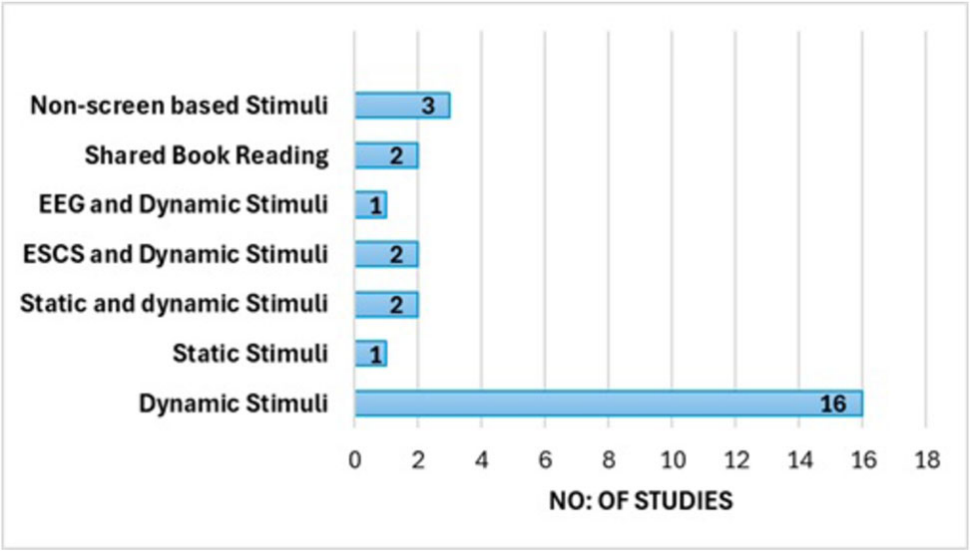
Categories and statistics of eye-tracking measures in the primary studies

- *Eye-tracking measures* JA, a vital social skill, relies on individuals establishing reference by following others’ gaze or directives like pointing (D’Entremont et al., 2017). Thus, analyzing gaze patterns and gaze following are integral to JA assessment (D’Entremont et al., 2017). Eye-tracking technology has emerged as a promising tool for this purpose, enabling the collection of eye movements and gaze patterns. It offers insights into social communication deficits, a key aspect of JA, by assessing metrics like eye contact duration and the frequency and direction of gaze movements (Jeyarani & Senthilkumar, 2023). Commonly used devices for capturing eye movements include SMI RED250, Tobii TX300, Tobii T60, T120, and Eye Tribe (Jeyarani & Senthilkumar, 2023). In most primary studies, JA assessment using eye-tracking technology is predominantly based on dynamic stimuli. In particular, 16 studies employed dynamic stimuli (Billeci et al., 2016; Chawarska et al., 2013; Falck-Ytter et al., 2012; Chawarska et al., 2012; Congiu et al., 2016; Caruana et al., 2018; de Belen et al., 2023; Falck-Ytter et al., 2015; Griffin & Scherf, 2020; Ozdemir et al., 2024; Parsons et al., 2017; Swanson & Siller, 2013; Temeltürk et al., 2023; Thorup et al., 2017; Vivanti et al., 2017; Zhang et al., 2022), while 2 studies used a mix of static and dynamic stimuli (Cilia et al., 2019; Gillespie-Lynch et al., 2013). Dynamic stimuli are represented by short video clips played for a few seconds, while static stimuli are still images. Figure 5 illustrates the difference between these two types of stimuli. Additionally, two studies combined the ESCS with dynamic stimuli (Franchini et al., 2017; Navab et al., 2012), and one study integrated EEG with dynamic stimuli (Billeci et al., 2017). Furthermore, two studies utilized shared book reading (SBR) as a stimulus (Ambarchi et al., 2024; Guo & Feng, 2013). The remaining studies employed various other approaches, including static stimuli (Riby et al., 2013), and non-screen-based stimuli (Ambrose et al., 2021; Nyström et al., 2017; Thorup et al., 2016). Brief descriptions of these studies are provided below and the statistics are shown in Fig. 6.
  - *Dynamic stimuli*: Parsons et al. (2019) analyzed the RJA using gaze-following patterns in high-risk ASD children using dynamic stimuli where an actress teaches word-object associations by shifting her head to an object with varying exclamation. Their findings indicated that infants who later developed ASD followed gaze as frequently as their TD peers, but spent less time engaged with either object. Additionally, it was observed that spending more time on faces and less on objects was linked to lower concurrent or subsequent verbal abilities, although it was not associated with later symptom severity. Falck-Ytter et al. (2015) and Vivanti et al. (2017) utilized the same JA task involving dynamic stimuli to assess gaze-following accuracy. In (Falck-Ytter et al., 2015), eye-tracking data were analyzed in intellectually low-functioning 3-year-olds with ASD. The results revealed that the duration of the first fixations at the objects in the scene indicated a notably weaker initial processing bias for attended objects in children with ASD in comparison to children with TD and non-autistic children with DD. Conversely, in Vivanti et al. (2017), children with ASD spent less time looking at the actor’s face and exhibited reduced gaze following when compared to TD participants. Quantifying gaze patterns during the observation of video clips depicting JA behavior enables the measurement of metrics such as the duration of fixation on a face (Chawarska et al., 2012, 2013) or the frequency of gaze shifts between a face and a referenced object in a JA interaction (Falck-Ytter et al., 2012). The study by Chawarska et al. (2013) examined spontaneous social monitoring in 6-month-old infants later diagnosed with ASD using eye-tracking. The task involved a 3-minute video of an actress engaged in various activities. Compared to TD and other high-risk infants, those later diagnosed with ASD showed significantly reduced attention to the overall social scene. In (Thorup et al., 2017), researchers investigated RJA using dynamic stimuli featuring an actress seated behind a table with four objects. The study aimed to determine if objects perceived as highly interesting by children with ASD would influence gaze-following behaviors. Results indicated no group differences in gaze following accuracy. Additionally, children with ASD showed shorter first fixation durations compared to the baseline condition, where ordinary objects were used. In a study similar to Chawarska et al. (2013), de Belen et al. (2023) examined RJA through gaze following in infants deemed at high-risk and low-risk for ASD, as well as ASD, and TD toddlers. During the eye-tracking experiment using the Tobii TX300 eye tracker, infants were observed following the experimenter’s gaze as they looked at objects to their left or right. Studies using dynamic stimuli (Caruana et al., 2018; Griffin & Scherf, 2020; Ozdemir et al., 2024; Swanson & Siller, 2013) found that children with ASD showed difficulties in gaze following compared to their TD counterparts. The study by Congiu et al. (2016) analyzed gaze-following responses in children with ASD. Eye-tracking was conducted using the Tobii T-60 eye tracker, employing dynamic stimuli. In the experimental setup, an actress presented an object, concealed it under one of two identical cups, shuffled the cups, directed her gaze toward the camera, and then looked toward the cup containing the object. The results revealed that ASD children were significantly less accurate in gaze-following and spent less time looking at the attended object. Billeci et al. (2016) used eye-tracking to take a deeper dive into the visual patterns of toddlers with ASD. Their study employed a dynamic eye-tracking paradigm with videos displaying male and female models shifting their gaze toward or away from an object. The researchers measured the proportion of fixations (PoF) on the object and face regions of interest (AOIs), including the eyes and mouth, to evaluate RJA and IJA abilities. The study revealed no differences between toddlers with ASD and TD toddlers in the RJA task, but significant differences in the IJA tasks. A recent study by Zhang et al. (2022) introduced RJAfinder, an automated tool for quantifying RJA behaviors in ASD using eye-tracking data. The RJAfinder was developed to create 12 video clips and used eye-tracking data recorded at a frequency of 300 Hz. The study found a negative correlation between RJA ability in ASD children and the Social Responsiveness Scale (SRS) scores, and a logistic regression model was built to classify ASD and TD children based on RJA events. The study provided an automated tool to quantify RJA, aiming to improve ASD screening, subtyping, and behavior interventions. The utility of eye-tracking in assessing JA deficits is not limited to ASD alone. Temeltürk et al. (2023) explored the use of eye-tracking to investigate autistic traits, RJA abilities, and face-scanning patterns in children with ADHD. They employed a dynamic eye-tracking paradigm similar to the one used by Billeci et al. (2016), measuring the PoF on the object and face AOIs to assess RJA. The outcomes revealed that children with ADHD had higher autistic traits and lower PoF on the object AOI, indicating RJA deficits compared to TD children.
  - *Static stimuli:* In (Riby et al., 2013), static stimuli were used to assess RJA capabilities in individuals with ASD. Eye-tracking was employed to investigate how participants attended to and interpreted an actor’s gaze cue within a social scene. Images were initially displayed for 3 s with instructions to simply observe the picture. The same images were then shown again, and participants were asked to identify the object the actor was looking at. The experiments revealed that individuals with ASD exhibited longer fixation durations compared to TD participants during gaze-following tasks.
  - *Static and dynamic stimuli:* The impact of static and dynamic stimuli was explored in Cilia et al. (2019), where the participants watched a series of photos (static) and a video (dynamic) intended to elicit JA behaviors. The authors employed Voronoi diagrams to determine the areas of interest (AOI), which were defined around the fixation densities of participants identified using a mean shift algorithm. Retroactive analysis of eye-tracking data was conducted to identify instances of RJA by examining participants’ actual fixations. The findings indicated that static stimuli facilitated the precise identification of AOIs and revealed similarities in visual exploration patterns between children with ASD and TD children. In contrast, differences in visual exploration were observed during dynamic stimuli, suggesting that the nature of the stimulus may influence JA behaviors differently across groups. Gillespie-Lynch et al. (2013) utilized static stimuli for gaze-cuing tasks and dynamic stimuli for world learning tasks in their study. JA bids were elicited through head and eye movements. The findings revealed that participants with ASD demonstrated diminished gaze-following abilities compared to TD children.
  - *ESCS and dynamic stimuli:* The study by Navab et al. (2012) examined the validity of using eye-tracking to assess RJA in 18-month-old siblings of children with autism. The researchers compared various eye-tracking measures of RJA to face-to-face assessments using the ESCS. They found that two eye-tracking measures – the standard difference score and percentage of accurate gaze shifts – were positively correlated with RJA during the distant pointing task of the ESCS, supporting the validity of the eye-tracking method. However, the eye-tracking measures were not related to RJA during the proximal book task of the ESCS. Additionally, most eye-tracking measures did not correlate with the infants’ language abilities or autism symptoms at 18 months, except for a negative correlation between one eye-tracking measure and social-affective autism symptoms. These findings suggest that while eye-tracking can provide a valid measure of some aspects of RJA, it may not capture all the complexities of JA behaviors observed in face-to-face interactions. The correlation of accurate gaze shifts with RJA on the ESCS was also explored in the study by Franchini et al. (2017). The researchers designed an eye-tracking task to measure RJA in response to gaze shifts with varying emotional intensities in both ASD and TD preschoolers. Conditions included gaze shifts with neutral, mild, or strong surprise expressions, as well as a pointing condition with a neutral expression. Results showed a decrease in overall time spent on communicative cues and a reduction in RJA in the ASD group compared to their TD counterparts.
  - *EEG and dynamic stimuli:* Recognizing the potential of combining multiple technologies, Billeci et al. (2017) conducted a pilot study that integrated eye-tracking with EEG to investigate the neural correlates of responding and initiating JA in high-functioning children with ASD. The researchers used eye-tracking measures to characterize the visual patterns associated with JA and then examined the corresponding brain activity and connectivity patterns using EEG. The results suggest the feasibility of using the proposed multimodal approach to characterize JA-related brain circuitries and visual patterns in ASD individuals and monitor longitudinal changes in response to interventions.
  - *Shared book reading (SBR):* Guo and Feng (2013) took the application of eye-tracking technology a step further by using it not only to assess but also to enhance JA in a different context: SBR between parents and children. The researchers conducted three experiments: a baseline measurement and two interventions where either children or parents received real-time feedback on their partner’s eye gaze. By simultaneously tracking the eye movements of both participants, the study measured JA in real time, defining it as moments when both partners looked at the same visual object on a page. Recently, Ambarchi et al. (2024) conducted a study investigating social and JA behaviors during SBR in young children with ASD, aiming to explore its potential as a marker for social development. The study involved four SBR videos where an adult reader engaged with a child while reading a children’s picture book, which included sequenced JA bids. Each JA bid was temporally segmented to reflect the three-sequence goal-directed process of an adult engaging with a child in shared awareness of the illustrative features in the picture book. The sequences were ordered as IJA, explicit cueing to the target object, and shared gaze between the adult reader and the child.
  - *Non-screen-based stimuli:* In the study by Thorup et al. (2016), infants were observed following the gaze of the experimenter as they looked at objects to their left or right. RJA was analyzed under two conditions, i) Eyes and Head Condition (involving turning both head and eyes) and ii) Eyes Only Condition (shifting gaze only without turning the head). The findings indicated that infants in the high-risk group were more likely to follow gaze in the Eyes and Head Condition compared to the Eyes-Only Condition. The study by Nyström et al. (2017) examined gaze patterns of 10-month-old infants at high risk for ASD and low-risk controls during a live interaction. Using eye-tracking technology, they recorded how infants responded when an adult looked directly at them while playing with a toy. The study found that high-risk infants looked less at the adult’s face specifically during the 300–1000-ms window after the adult initiated eye contact. Importantly, this difference was only detectable through precise eye-tracking measurements and was not apparent in overall looking times at the face. The study by Ambrose et al. (2021) involved a pilot eye-tracking study in a guided interaction between a 9-year-old boy with ASD and occupational therapists. The study utilized SensoMotoric Instruments (SMI) ETG 2w mobile eye trackers and pan-tilt-zoom (PTZ) cameras to analyze interactive behaviors such as mutual face gaze (MFG) and IJA during a tabletop block construction task. The experimental task involved building animal constructions (a dinosaur with 66 blocks and a spider with 63 blocks) using step-by-step pictorial instruction guides. The child completed one construction with each therapist, who used different interaction styles. The interactions were video recorded from multiple perspectives, and eye-tracking data was analyzed using defined AOIs including the partner’s head, torso, hands, the build area, instructions, and blocks. The results indicated differences in gaze use and interactive JA behaviors between the therapists working with the child and within the child’s dyadic interactions.
- *Virtual reality (VR) platforms* VR platforms are software and hardware systems designed to create immersive, interactive, and three-dimensional environments that users can explore and interact with. It accomplishes this by integrating stimuli across various sensory channels – like vision, auditory cues, and tactile feedback – alongside motion tracking and control mechanisms, including force feedback devices (Parsons et al., 2017). Research in JA has also seen significant advancements in the use of VR platforms. These VR-based systems offer a controlled and highly interactive environment to assess and train JA skills in individuals with ASD (Jyoti & Lahiri, 2019). Of the nine primary studies identified, seven utilized virtual characters or avatars for JA tasks (Amaral et al., 2018; Amat et al., 2021; Caruana et al., 2017; Jyoti & Lahiri, 2019; Little et al., 2016; Mundy et al., 2016; Pfeifer-Lessmann et al., 2012). In addition, one study employed a head-mounted VR system (Ravindran et al., 2019), while another developed a VR tool to validate the C-JARS assessment (Yazdanian et al., n.d.). The tasks in (Caruana et al., 2017; Pfeifer-Lessmann et al., 2012), involve gaze-based cueing achieved by altering the looking direction and adjusting the pointing direction through the eyes of virtual characters. Viewing times and recognition memory for the referent images were measured and compared between conditions involving RJA and IJA. Gaze cueing with a VR avatar by Mundy et al. (2016) was motivated by the theory that atypical information processing during JA is a feature of ASD, which may impact learning and social cognition. Children participated in two blocks of 12 RJA picture study trials and two blocks of 12 IJA trials, totaling 24 pictures for each type. In the RJA condition, children were instructed to make eye contact with the avatar and then follow the avatar’s gaze direction to the left or right to view a picture for one second. On the other hand, in the IJA condition, children chose which side of the avatar to look at, after which pictures would appear, and the avatar would follow the child’s line of regard. Amaral et al. (2018) conducted a feasibility clinical trial on social attention, utilizing a virtual environment with avatars by measuring JA cues. They developed a customized JA assessment task to detect IJA cues from pointing or avatars’ gaze. A VR-based JA task (ViJAT) platform with a desktop VR setup was developed by Jyoti and Lahiri (2019). Within this platform, virtual characters presented JA cues at various hierarchical levels, including eye gaze, head turn, finger pointing, and sparkling cues, adjusting based on the participant’s performance. The system quantitatively measured JA skills through a scoring mechanism where correct responses at earlier cue levels received higher scores (e.g., 100 for eye gaze, and 70 for head turn). The study by Amat et al. (2021) presented a similar VR-based system called InViRS, which also incorporated hierarchical JA cues in a virtual environment. The InViRS platform allowed for adaptive task difficulty based on individual performance and provided quantitative estimates of JA skills. These estimates were obtained through real-time eye tracking to measure gaze fixation on the avatar’s face and eyes, game performance metrics like score, completion time, and response time to gaze prompts, and assessment of gaze following ability at different prompt speeds. Ravindran et al. (2019) explored the feasibility and usability of using Floreo’s VR platform to support JA skills in school-aged children with ASD. Floreo’s VR platform is a specialized system designed to deliver immersive social and behavioral interventions for individuals with ASD through head-mounted displays (Ravindran et al., 2019). A recent study by Yazdanian et al. (n.d.) evaluated JA using a virtual cross-validation tool for the C-JARS assessment method. This tool incorporated a series of VR tasks called JA Assessment Tasks (JAST), which involved interacting with an avatar in a virtual living room.
- *Robotic systems* A robotic system for JA assessment involves the use of robots or robotic devices to interact with individuals and evaluate their JA skills. These systems usually employ robots equipped with sensors, cameras, and programmed behaviors to engage in social interactions with users. During these interactions, the robot may use cues such as eye gaze, gestures, or verbal prompts to elicit JA behaviors from the individual being assessed (Zheng et al., 2020). The robot’s responses and the individual’s reactions are then observed and analyzed to assess their JA abilities. Single humanoid social robots (David et al., 2018; Kumazaki et al., 2018; Zheng et al., 2020) were used in three primary studies, whereas one study (Mehmood et al., 2019) employed a multi-robot system for assessment. Zheng et al. (2020) used the commercially available NAO humanoid robot (Robotics, n.d.), which provided JA prompts with two computer monitors serving as attentional targets. The robot’s prompts were automatically adapted based on the child’s real-time performance, tracked by cameras. JA assessment included both a human-administered STAT (Screening Tool for Autism in Toddlers and Young Children) to evaluate generalization to human interactions and within-system measurements of the child’s responses to the robot’s prompts. In the study by Kumazaki et al. (2018), the robot Commu, along with a human agent, was employed to improve JA skills in children with ASD. JA cues were initiated by either the human agent or Commu through gaze shifts toward the child and then toward images placed on the left and right sides. The participant’s RJA was measured by tracking head turns and gaze direction. Expanding on this, David et al. (2018) examined the influence of different social cues (gaze, pointing, and vocal instruction) used by a robot or human partner in a single-case study design. JA’s performance was measured using a behavioral grid that assessed the child’s response (head-turning, pointing, or vocalization) to the partner’s cues across three conditions: gaze only, gaze + pointing, and gaze + pointing + vocal instruction. Children had multiple opportunities to respond in each condition, with higher scores indicating better JA performance. The study by Mehmood et al. (2019) conducted a novel investigation into the distribution of JA and imitation skills in children with ASD using a multi-robot system. The researchers employed two humanoid robots positioned one meter apart, with integrated JA and imitation modules. Participants’ interactions with the robots were analyzed for JA initiation, imitation accuracy, and eye contact patterns. EEG recordings were also used to assess brain hemisphere dominance.
- *Vision-based methods* In addition to the aforementioned technologies, computer vision techniques have also been explored for detecting and assessing JA skills in individuals with ASD. While cameras were primarily used to record JA assessment sessions for subsequent video coding, the use of videos to develop AI models remains underexplored. Consequently, only three studies were identified that employed computer vision and AI for automated assessment. Zhang et al. (2018) proposed a vision-based system that uses an RGB camera and Kinect sensor to detect JA automatically. The key components of their system include hand gesture recognition, face detection, and eye gaze tracking, which are used to measure the essential elements of JA – the adult’s pointing/gaze, eye contact between the adult and child, and the child’s gaze following the adult’s to the target object. The researchers evaluated their system by testing it on eight non-ASD adults and found that it could effectively detect JA with good accuracy, except for one case where the child’s small face size led to poor eye gaze detection. Similarly, Prakash et al. (2023) assessed a broader range of behaviors and skills in children with ASD, including JA using computer vision. In particular, they developed two models – one to recognize when a child follows the gaze direction of the therapist to establish JA, and another to detect when the child points a finger to respond to the therapist’s verbal questions and establish JA. Another method proposed by Ko et al. (2023) focused on JA tasks, video data acquisition, and a deep learning (DL) model to detect and assess symptoms of ASD. The JA-eliciting protocol was administered, and video data were collected for diagnostic purposes. This protocol involved three types of JA tasks: IJA, low-level RJA, and high-level RJA. For example, in the IJA task, children were presented with a toy to trigger social interaction initiation. The study’s innovative approach involved the creation of a DL classification system to process the video data. Furthermore, the study utilized explainable AI techniques to visualize the DL system’s decision-making process and interoperability. These techniques revealed that features most correlated with JA included triadic gaze patterns (shifting gaze between object and examiner) in TD children during IJA tasks, sustained gaze on presented objects in TD children during low-level RJA tasks, and immediate head turning to view distant stimuli in TD children during high-level RJA tasks. In contrast, children with ASD showed a lack of these behaviors or delayed responses.

#### Methodological insight and rationale for approach selection

The primary studies discussed in this section select JA assessment approaches based on their research objectives, target population, and practical constraints. Human-mediated methods (e.g., ESCS, ADOS, play-based observations) are often preferred when rich contextual understanding, developmental appropriateness, and clinical interpretability are top priorities, particularly in diagnostic settings involving young children. These approaches allow flexible prompting and capture fine differences but are limited by subjectivity, scalability, and observer bias. In contrast, technology-assisted methods are increasingly adopted when objective, fine-grained, and reproducible measurements are required, especially in large-scale studies or longitudinal monitoring. Eye-tracking is commonly chosen for its precision in quantifying gaze-following (RJA), while VR and robotic systems are favored when experimental control and standardized social stimuli are needed. Vision-based and AI-driven approaches are typically selected to enhance automation and ecological validity in real-world settings. Hybrid approaches emerge when studies aim to balance ecological richness with objectivity, leveraging the complementary strengths of human judgment and automated measurement. In addition, Table 5 provides a comparative summary of the key characteristics, methodological overlaps, contrasts, and the growing role of combined approaches that integrate the ecological validity of human-mediated approaches with the objectivity and scalability of technology-assisted methods.

**Table 5:** Comparative summary of human-mediated, technology-assisted, and combined approaches for JA assessment

Dimension	Human-mediated	Technology-assisted	Combined
Definition	Direct observation-based assessment conducted by clinicians, researchers, caregivers, or educators	Assessment using digital technologies, sensors, or AI-driven systems	Combination of human-mediated methods with technology-assisted tools
Methods	ESCS, ADOS, JA Protocols, play-based assessment, parental surveys (CSBS-DP, C-JARS)	Eye-tracking, VR, robotic systems, vision-based and AI-based methods	ESCS/ADOS combined with eye-tracking, vision-based analysis, or VR systems
Behaviors assessed	IJA, RJA, joint engagement	Predominantly RJA; increasing assessment of IJA in vision-based and VR approaches	IJA and RJA assessed concurrently
Measurement nature	Qualitative and semi-quantitative behavioral coding	Quantitative, fine-grained gaze, motion, or interaction metrics	Behavioral coding supported by objective technological measures
Objectivity	Dependent on rater expertise and scoring protocols	Automated or semi-automated measurements	Reduced subjectivity through technological support
Ecological validity	High in naturalistic and play-based settings	Lower in screen-based eye-tracking, higher in VR, robotics, and vision-based systems	Preserves social interaction while improving measurement precision
Scalability	Time-intensive and requires trained personnel	Suitable for large-scale and longitudinal assessment	Dependent on integration complexity and resources
Clinical relevance	Widely used in clinical diagnosis and intervention planning	Primarily research-oriented, emerging clinical applications	Supports translational and validation-focused research
Resource requirements	Training and administration time	Specialized hardware, software, and expertise	Requires both clinical expertise and technological infrastructure
Key strengths	Context-rich, developmentally sensitive, well-validated tools	Precision, reproducibility, fine temporal resolution	Balanced assessment combining ecological validity and objectivity
Key limitations	Subjectivity, inter-rater variability, limited temporal resolution	Reduced social richness in some setups; cost and accessibility	Increased methodological and analytical complexity
Typical study contexts	Clinical assessment, early screening, small- to medium-scale studies	Experimental studies, large-scale screening, automated analysis	Validation studies, and real-world deployment
Emerging trends	Increased focus on naturalistic and classroom-based assessment	Shift toward computer vision, multimodal sensing, and AI-based analysis	Growing adoption to overcome limitations of single-method approaches

### RQ2-target groups

The 28 primary studies employing human-mediated methods for JA assessment focused on diverse target groups. The sample size of the participants ranges from 18 to 2000. All studies consist of ASD participants except 3, with one targeted TD and the other for intellectual disabilities (ID). Ten studies exclusively targeted individuals with ASD, while nine studies included both TD individuals and those with ASD. Two studies focused on younger siblings of TD and ASD children. Additionally, three studies included a broader range of participants, comprising individuals with ASD, TD, and DD. One study examined participants with ASD, ID, and TD individuals, while another focused on individuals with ASD and DD. Furthermore, one study explored JA assessment among individuals with ASD and Fragile X syndrome (FXS).

The human-mediated methods for JA assessment primarily focused on infants (0–12 months) and toddlers (1–3 years). This emphasis is rooted in the critical role of JA as an early developmental marker for ASD. Early identification of JA deficits is crucial since timely and appropriate interventions can significantly improve developmental outcomes in children at risk for ASD. During infancy and toddlerhood, children are naturally developing foundational social-communication skills, making these age groups ideal for detecting early signs of atypical behavior, including difficulties in establishing and RJA cues. Although the primary focus was on younger age groups, school-aged children (5–16 years) were also included in some studies. The assessment for this age group is typically aimed at evaluating JA skills that may have either developed naturally or improved through previous interventions. Additionally, this age group allows researchers to assess whether deficits persist and how they affect social interactions and academic engagement over time. The inclusion of school-aged children also provides valuable insights into the longitudinal development of JA skills and their role in social and educational contexts.

**Fig. 7:**
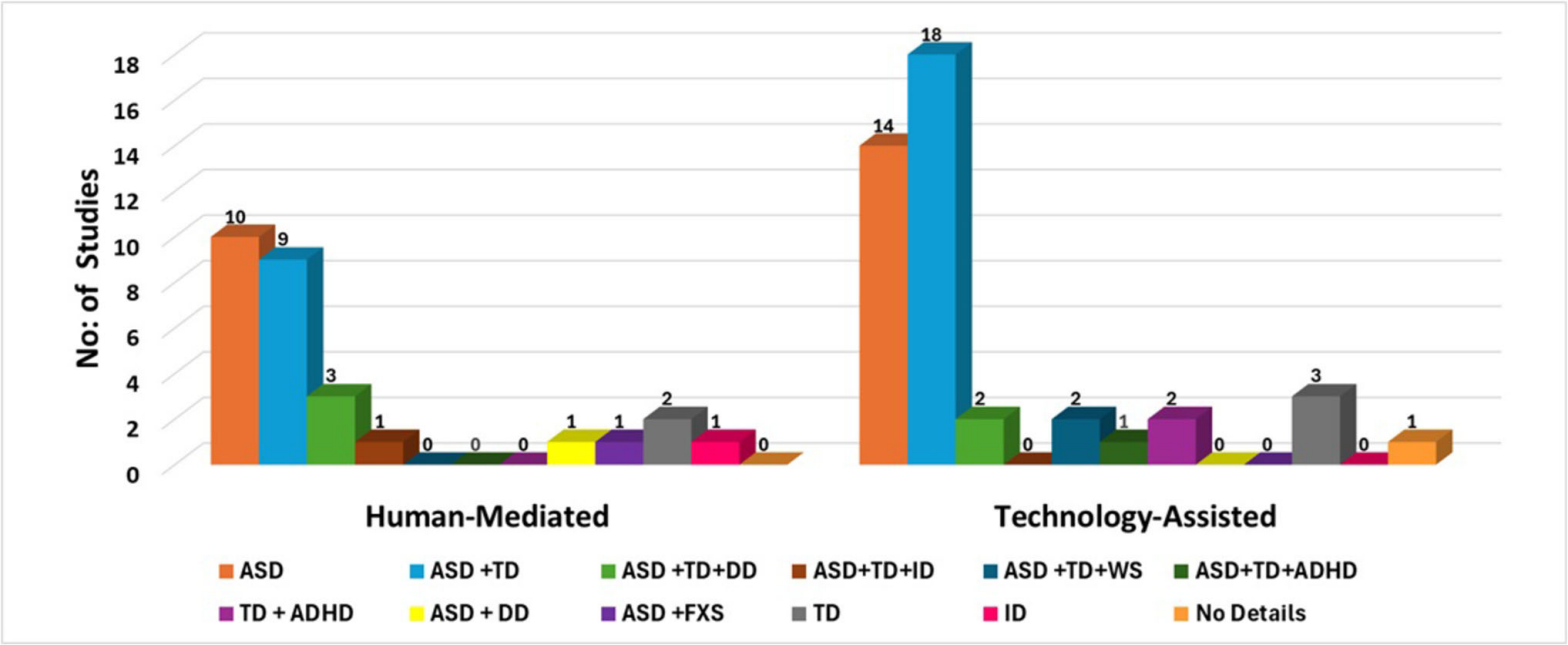
Statistics of target groups in human-mediated and technology-assisted approaches in the primary studies

In technology-assisted approaches, similar to human-mediated methods, the primary focus was on children with ASD and their TD counterparts. Infants were predominantly considered in eye-tracking studies, while approaches involving VR, robotics, and computer vision techniques targeted toddlers, preschool children, and adults. Out of 43 primary studies employing technology-assisted approaches, 18 included both ASD and TD participants, while 14 focused exclusively on individuals with ASD. Additionally, participants with ADHD and Williams syndrome (WS) were included in two studies each. Three studies focused solely on TD children, and one study did not specify participant details. The sample sizes across these studies ranged from 1 to 143 participants. Figure 7 provides the statistics of target groups used in the primary studies.

### RQ3- Measures for assessment and outcomes

#### Measures for assessment

**Fig. 8:**
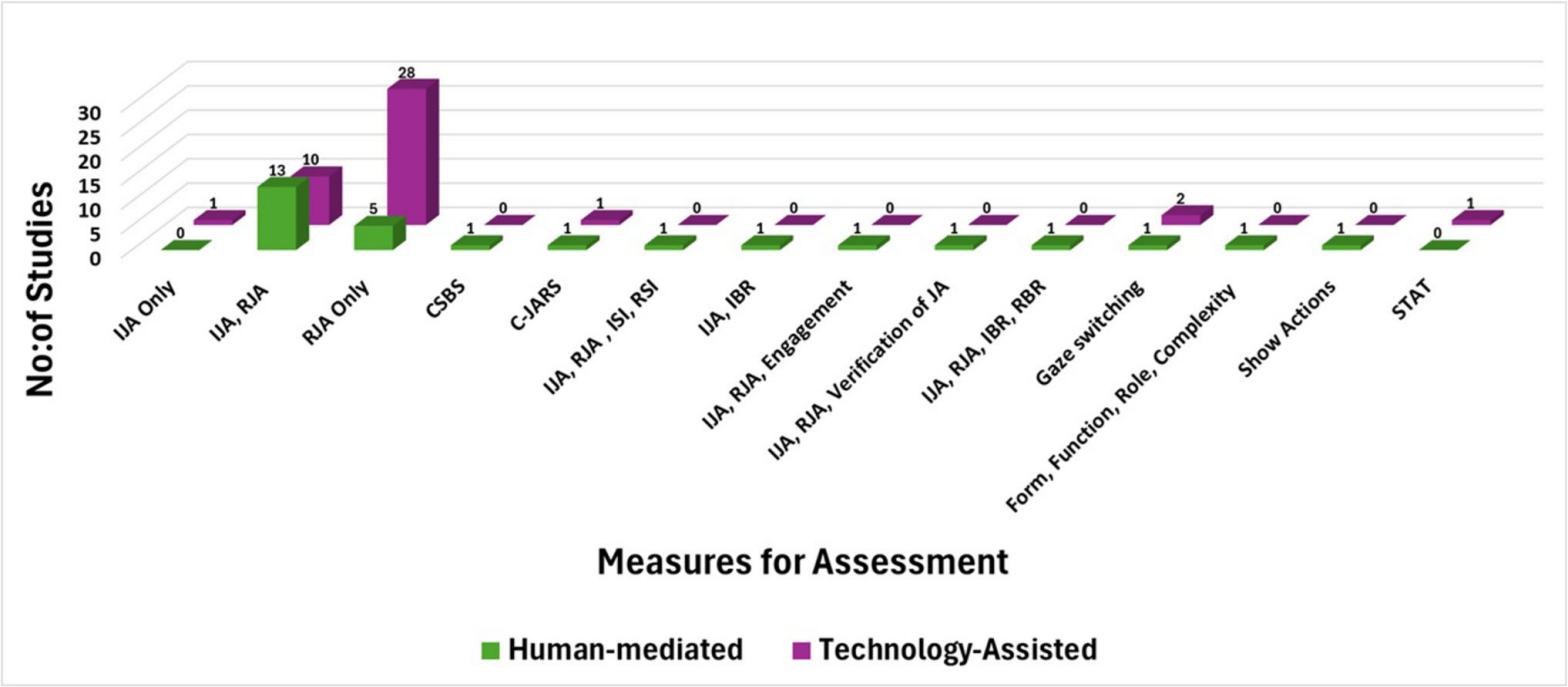
Comparison of various measures used in the primary studies for JA assessment in human-mediated and technology-assisted approaches

The primary studies commonly assess JA using expressive and receptive behaviors, specifically IJA and RJA. In human-mediated approaches, both IJA and RJA are frequently utilized (*n* = 13), while RJA emerges as the predominant measure in technology-assisted approaches (*n* = 28). Figure 8 presents a comparative overview of the number of studies employing these measures across both approaches. In the category of IJA, no studies employed human-mediated assessments, whereas one study used a technology-assisted approach. This highlights the potential for technological solutions in tasks where detecting gaze cues and spontaneous initiations are critical. Similarly, for RJA, technology-assisted assessments (28 instances) far outnumbered human-mediated ones (five instances). This trend can be attributed to the ability of automated systems to efficiently and precisely track gaze direction and detect attention responses.

Some categories, such as CSBS and C-JARS, show a balanced or human-centered approach. CSBS, a standardized tool, appears to be predominantly used in human-mediated setups, while C-JARS demonstrated equal adoption in both methodologies. The assessment of complex communicative functions and roles and behavioral dimensions like IJA, RJA, engagement, and behavioral requests (IBR and RBR) relied mostly on human-mediated methods, likely due to the qualitative and subjective nature of these tasks. Interestingly, technology-assisted methods gained an advantage in tasks involving gaze switching and objective evaluations such as the STAT. This reflects the strength of technology-assisted approaches in precisely capturing and analyzing visual and spatial cues. However, tasks like verification of JA, engagement analysis, and behavioral assessments are still predominantly human-mediated, indicating the complexity and contextual understanding required, which machines struggle to handle.

#### Outcomes

The outcomes of the primary studies are categorized based on the following aspects targeted in the assessments:

- *Early identification:* The study in Montagut-Asunción et al. (2022) conducted a longitudinal study involving 32 infants assessed at two developmental stages (8 and 12 months) to investigate whether their JA skills were associated with early signs of ASD at 18 months. Logistic multiple regressions were employed for data analysis, revealing that higher IJA at 8 months and RJA at 12 months were correlated with an increased risk of ASD at 18 months, highlighting the crucial role of early JA skills in identifying early manifestations of ASD. Charman (2003) found that JA behaviors during the active toy task were associated with later language and symptom severity, emphasizing the pivotal role of JA in the development and severity of ASD symptoms. Moreover, Sullivan et al. (2007) discussed the importance of RJA as an early marker and target for intervention in ASD.
- *Social communication:* The findings in Charman (2003) revealed that greater proficiency in gaze switching during active toy tasks at 20 months was linked to less severe social and communication symptoms at 42 months. The authors of Wetherby and Prizant (2002) discuss the importance of early identification of communication delays to enable early intervention, which has a greater impact on outcomes. The parent-report measure, C-JARS (Mundy et al., 2017), aimed at evaluating JA skills in children, taking into account verbal behavior. Initial findings suggest that symptoms associated with reduced JA and the spontaneous sharing of experiences with others can be evaluated using this measure in children and adolescents with ASD. The C-JARS is noted to assess a distinct aspect of the social characteristics of children with ASD. On the other hand the SCATA (Drew et al., 2007) to measure the frequency, form, and function of communication in toddlers with ASD, with scoring dimensions consisting of form, function, role, and complexity of communicative acts. Results from Franchini et al. (2017) show a decrease in overall time spent on communicative cues and reduced RJA in the ASD group compared to their TD counterparts. Additionally, these eye-tracking variables correlated with adaptive behavior, particularly in the Communication domain, as assessed by the Vineland Adaptive Behavioral Scale, 2nd Edition (Sparrow & Cicchetti, 1985). The study by Zhang et al. (2022) found a negative correlation between RJA ability in ASD children and the SRS scores.
- *JA initiations and responsiveness:* In (Paparella et al., 2011), timing and sequence of JA during IJA and requesting skill acquisition during RJA were analyzed in children with ASD to check whether it differ from those in TD children. The study employed both cross-sectional and longitudinal samples. Results revealed notable differences in the acquisition sequence of JA skills between children with ASD and TD children. Particularly, the skill of following gaze, which emerged approximately mid-sequence in TD, was observed as the last nonverbal form to emerge in children with ASD. Additionally, the skill of showing emerged sequentially later than in the typical group. In addition, Cilia et al. (2020) found that ASD children were less responsive than TD children. The study also highlighted the developmental progression in the responses of children with ASD when multiple deictic cues (pointing and verbalizations) were used by an adult simultaneously, whether the referent was present or absent from the child’s visual field. The type of cues used by the adult was found to affect ASD children’s response behaviors during JA towards a referent. MacDonald et al. (2006) found that children with autism had relatively minor deficits in RJA compared to TD children, but more severe deficits in IJA, with over half the children with autism having low or no JA initiations. The study in toddlers by Billeci et al. (2016) revealed no differences between toddlers with ASD and TD toddlers in the RJA task, but significant differences in the IJA tasks. Key findings from Nyström et al. (2019) indicated that at 10 months, infants who were later diagnosed with ASD exhibited significantly lower rates of IJA compared to the other groups, but their rates improved by 18 months. In contrast, RJA did not differentiate the ASD group, although TD infants had higher overall RJA scores than the combined high-risk groups. The authors emphasize the potential of live eye-tracking to detect early gaze atypicalities in ASD and underscore IJA as a crucial focus for future early intervention efforts. In (Mundy et al., 2016) TD and ADHD groups displayed significantly better recognition memory for pictures studied in the IJA condition compared to the RJA condition. However, this effect was not evident in the ASD group.
- *Language development:* The correlation between JA and expressive language development was examined in Adamson et al. (2019); Chiang et al. (2008), and in Abdelaziz et al. (2025), and the outcomes reveal that toddlers identified as having a heightened risk of ASD, particularly those later diagnosed with ASD, exhibited weaker JA abilities, joint engagement during parent-toddler interactions, and expressive language skills. These results further emphasize the interconnected nature of JA and language development. In (Maljaars et al., 2012), the study included three groups: children with ASD and ID, children with ID, and TD children. The results revealed notable distinctions in language skills across the three groups, with decreased levels of JA showing a robust correlation with concurrent language proficiency in low-functioning children with ASD. The relation between JA abilities and language skills was also explored in Thurman and Dimachkie Nunnally (2022), focusing on children diagnosed with ASD and Fragile X syndrome. Their findings revealed robust correlations between the overall JA scores and language measures among boys with autism. Furthermore, JA performance exhibited a negative correlation with autism symptom severity in both groups, and among boys with autism, it was also negatively associated with symptoms of ADHD. However, no significant correlations were observed between JA scores and language measures in boys with Fragile X syndrome. Additionally, it was observed that spending more time on faces and less on objects was linked to lower concurrent or subsequent verbal abilities Parsons et al. (2019).
- *ASD symptom:* Parsons et al. (2019) found that infants who later developed ASD followed gaze as frequently as their TD peers but spent less time engaging with objects. Less accurate gaze following was linked to more severe ASD symptoms (Parsons et al., 2019), highlighting significant differences in how high-risk and low-risk infants respond to social cues like eye contact. Additionally, the duration of initial fixations on objects revealed a weaker processing bias for attended objects in children with ASD compared to TD and non-autistic children with DD (Falck-Ytter et al., 2012; Vivanti et al., 2017). Similarly, children with ASD in Swanson and Siller (2013) spent less time looking at the actor’s face and demonstrated reduced gaze-following behavior compared to TD participants. Ko et al. (2023) DL models demonstrated high accuracy, precision, and recall in identifying ASD and differentiating levels of symptom severity, with IJA-based models outperforming RJA-based models in severity assessment. Using explainable AI techniques like gradient-weighted class activation mapping and attention plots, the study visualized the decision-making process, showing that triadic gaze patterns in TD children during IJA tasks, sustained gaze on objects in low-level RJA tasks, and immediate head turning during high-level RJA tasks were key features, while children with ASD lacked these behaviors or displayed delayed responses.
- *Social and cognitive abilities:* The study in Schietecatte et al. (2012) investigated the characteristics of JA deficits in children with ASD at the age of 36 months, focusing on social and cognitive abilities like attention disengagement, social preference, and intention understanding. Their findings revealed that RJA correlated with intention understanding, while the frequency of JA initiations was linked to attention disengagement and, to a slightly lesser extent, social preference. Bean and Eigsti (2012) found that the JA measure effectively discriminated between ASD and TD groups, and showed associations with receptive language, social ability, and theory of mind in the ASD group. This assessment method offers a way to measure JA skills in older children and adolescents, addressing a gap in existing measures that are primarily designed for younger children. The study by Chawarska et al. (2013), examined spontaneous social monitoring in 6-month-old infants later diagnosed with ASD using eye-tracking. Specifically, Infants with ASD spent on average 33.7% of time looking at the scene, compared to 40.7% for high-risk atypical infants, 39.1% for high-risk typical infants, and 42.5% for low-risk typical infants (p = .001). When examining attention to the person, infants with ASD devoted 52.4% of their looking time to the person, compared to 58.5%, 58.7%, and 57.6% for the other groups, respectively (*p* = .018). For attention to the face, infants with ASD spent 18.5% of looking time on the face, versus 23.4%, 26.9%, and 23.5% for the comparison groups (*p* = .009). These quantitative results demonstrate that by 6 months of age, infants later diagnosed with ASD already show measurable reductions in visual attention to social stimuli across multiple levels of analysis. The findings in Thorup et al. (2016) and de Belen et al. (2023) indicated that infants in the high-risk group were more likely to follow gaze. Moreover, de Belen et al. (2023) identified a correlation between higher accuracy in gaze following and better early cognition and adaptive behaviors in children with ASD.
- *Naturalistic observation:* The findings by Roos et al. (2008) demonstrated strong positive correlations between the frequencies and proportions of JA instances across standardized coding and naturalistic observation. The ESCS notably elicited a greater number of initiated JA episodes, while the play sample captured more nuanced responses to JA bids. This alignment supports the idea that naturalistic sampling can serve as a valuable alternative or complement to standardized measures. The analysis by Panganiban (2017) indicates that structured and unstructured play interactions are effective in measuring child-initiated JA skills, with stronger correlations between measures for IJA and IBR. Furthermore, observing children with autism and those with other DD in preschool classrooms, the study (Wong & Kasari, 2012) found that children with autism spent more time unengaged and exhibited lower levels of symbolic play and JA compared to their peers.
- *Verbal and nonverbal intelligence:* The study by Harrison et al. (2016) aimed to assess the relationship between JA and verbal IQ (VIQ) and non-verbal IQ (NVIQ), utilizing the Differential Ability Scales – Second Edition (DAS-II) (Elliott et al., 1990) or the Mullen Scales of Early Learning [47]. The findings indicated that VIQ fully mediated the relationship between JA and adaptive functioning, and was partially mediated by NVIQ. The results in Sano et al. (2021) revealed a significant association between severe JA deficits and low intelligence, with greater severity of JA deficits corresponding to lower scores in problem-solving ability and knowledge of things. The study suggested that these specific relations might drive the overall relationship between JA and intelligence in children with ASD. They utilized the Japanese version of the Kaufman Assessment Battery for Children to assess intelligence (Kaufman et al., 1987). item *Visual engagement:* The study in Thorup et al. (2016) aimed to determine if objects perceived as highly interesting by children with ASD would influence gaze following behaviors. Results indicated no group differences in gaze-following accuracy, and ASD participants showed shorter first fixation durations than in the baseline condition with ordinary objects (Congiu et al., 2016; Thorup et al., 2016). Results from Congiu et al. (2016) also showed that ASD children were significantly less accurate in gaze-following and spent less time looking at the attended object. A retrospective analysis of eye-tracking data was conducted (Cilia et al., 2019) to identify instances of RJA by examining participants’ actual fixations. The results showed that static stimuli helped accurately identify AOIs and revealed similar visual exploration patterns between children with ASD and TD children. However, differences in visual exploration emerged with dynamic stimuli, indicating that the type of stimulus may influence JA behaviors differently across groups. In their study, Gillespie-Lynch et al. (2013) used static stimuli for gaze-cuing tasks and dynamic stimuli for world-learning tasks, finding that children with ASD exhibited reduced gaze-following abilities compared to TD children. In a study by Billeci et al. (2016), toddlers with ASD exhibited atypical gaze patterns, such as longer looking times at the face, more transitions from the target object to the face, and fewer transitions between objects compared to their TD peers. Children with ADHD had higher autistic traits and lower point of fixation (PoF) on the object AOI, indicating RJA deficits compared to TD children (Temeltürk et al., 2023). Additionally, children with ADHD exhibited higher PoF on the face and eye AOIs, suggesting atypical face scanning patterns. However, the study found an inverse association between autistic traits and PoF on the face AOI, irrespective of the ADHD diagnosis. The study by Guo and Feng (2013) tracked the eye movements of ASD and TD participants to measure JA in real-time, defining it as moments when both individuals focused on the same visual object. Results showed significant improvements in JA and word learning for both the child intervention (from 5.35% to 22.7%, learning 1.0 words) and parent intervention (from 3.48% to 12.87%, learning 1.25 words), outperforming the baseline where JA only increased slightly and word learning was minimal.
- *Executive function:* The studies by Lasch et al. (2023) using DJAA scores reveal that RJA is associated with executive function. Their findings suggest that higher RJA performance correlates with better cognitive control and decision-making abilities, which are key components of executive function.
- *Social interaction:* Kidwell and Zimmerman (2007) illustrated that JA is not solely a psychological process but fundamentally an interactional one embedded within social interactions. The study provides a detailed analysis of how young children engage in practical actions, such as showing objects to others, to capture and maintain attention. It emphasizes that JA involves more than simply co-orienting to an object; it includes the critical act of communicating the social intent behind the interaction. By employing a "proof procedure" to make each participant’s understanding observable, the study highlights JA as a socially constructed achievement that children adeptly manage with an awareness of social dynamics.

### RQ4-Open research issues and future directions

#### Open research issues

The human-mediated qualitative methods discussed in this review range from structured and semi-structured to unstructured and hybrid forms of assessments. While they quantify JA using standardized and psychological approaches, they require trained personnel to determine the level and nature of JA, introducing a degree of subjective interpretation. Additionally, most studies employ small sample sizes, making it difficult to assess the relationship between JA and functional outcomes in larger populations. While they provide valuable insights into a child’s ability to engage in JA, these assessments are often time-consuming and require extensive training to administer reliably. Moreover, their reliance on subjective judgment can introduce variability and potential bias in the results.

On the other hand, recent technological advancements have introduced more objective and scalable approaches to JA assessment. Eye-tracking technology, for example, provides precise measurements of gaze patterns and shifts, enabling researchers to quantify JA behaviors with high accuracy. Studies utilizing eye-tracking have demonstrated its effectiveness in identifying subtle deficits in JA that traditional methods might miss. However, eye-tracking systems can be expensive and may not be readily accessible in all clinical settings.

VR is emerging as an innovative tool for JA assessment. A VR environment can simulate social interactions and dynamically manipulate social cues, offering a controlled yet immersive platform for evaluating JA. Preliminary studies suggest that these technologies can engage children in JA tasks more effectively than traditional methods (Amat et al., 2021). However, the feasibility and practicality of implementing VR in routine clinical practice remain to be fully explored.

Similarly, robotic systems and vision-based algorithms have shown promise in automating JA assessment. These approaches can provide continuous, real-time data on head and body orientation, enabling the detection of JA behaviors in naturalistic environments. Despite their potential, these technologies are still in the developmental stage, and further research is needed to validate their accuracy and reliability in diverse populations.

#### Future directions

This section discusses new directions that have yet to be explored in the assessment of JA. These directions are derived from the insights, observations, and open research issues identified in existing approaches.

- *Fusion of multimodal data:* One promising direction in JA assessment is the fusion of multimodal data, which involves combining behavioral observations with visual, audio, and eye-tracking data. This multimodal approach presents a more robust, dense, and complete representation of behavior for several reasons. Firstly, different sensing technologies excel at capturing particular aspects of a phenomenon. For instance, visual data can provide detailed information on facial expressions and body movements, audio data can capture nuances in vocal intonations and speech patterns, and eye-tracking data can precisely measure gaze direction and fixation durations. Secondly, multimodal sensing captures a broader range of information, effectively overcoming the limitations inherent in relying on a single sensing modality (Varghese et al., 2021). Incorporating AI into this multimodal approach demonstrates remarkable potential in utilizing a wide array of data to solve real-world problems, particularly in the context of JA assessment (Porayska-Pomsta et al.., 2018). AI algorithms can analyze and integrate large amounts of data from multiple sources, uncovering patterns and correlations that might be missed by human observers or single-modality analyses. For example, AI can synchronize visual data with eye-tracking information to determine whether a child’s gaze direction aligns with their facial expressions during social interaction, providing insights into their JA capabilities. Moreover, the accuracy of AI-driven predictions may vary depending on the types and sources of data used. High-quality data from reliable sensors enhances the predictive power of AI models. For instance, combining high-resolution video recordings with precise eye-tracking data can lead to more accurate assessments of a child’s ability to follow gaze or point to shared objects (Varghese et al., 2024). The fusion of multimodal data also enables the development of personalized intervention strategies. By capturing a detailed and nuanced picture of a child’s JA abilities, clinicians can tailor interventions to address specific deficits. For example, if a child demonstrates good eye contact but struggles with following gestures, targeted therapies can focus on improving their response to non-verbal cues. Additionally, continuous monitoring using multimodal data can track the child’s progress over time, allowing for dynamic adjustments to their intervention plan. Furthermore, the integration of advanced technologies such as VR and robotics into multimodal JA assessment opens new avenues for creating engaging and controlled environments for children. VR scenarios can simulate real-world social interactions in a safe and controlled setting, providing consistent and repeatable conditions for assessment (Parsons et al., 2017). Social robots equipped with sensors can interact with children, offering a novel way to measure JA and other social skills. These technologies, combined with AI, can create immersive and interactive experiences that are both enjoyable for children and informative for researchers. An additional benefit of fusing multimodal data with AI is the potential to significantly reduce testing time. Traditional observational studies often require extended periods, sometimes exceeding 30 min, which can lead to restlessness in children and fatigue in examiners (Zhang et al., 2018). New technological approaches have the potential to assess the same attributes using various sensing methods and AI analysis, potentially decreasing the overall duration of testing sessions. This increased efficiency not only improves the experience for both children and examiners but also allows for more frequent assessments. Moreover, the time-saving aspect of these advanced technologies extends beyond just the assessment of JA. The integration of VR, robotics, and AI can potentially streamline the evaluation of a broader range of social and cognitive skills, offering a more holistic and time-efficient approach to developmental assessments.
- *Streamlining task complexity in assessments:* Current clinical approaches for JA assessments utilize numerous tasks, many of which are complex and demand highly reliable measurements (Lee et al., 2018). These tasks often include a range of activities designed to evaluate an individual’s ability to coordinate attention with another person. For example, tasks may involve following a caregiver’s gaze, pointing to objects, or engaging in interactive play. However, the validity of these attention tasks can vary significantly between pre-test and post-test evaluations, leading to inconsistencies that complicate the assessment of performance improvements from interventions (Porayska-Pomsta et al.., 2018). This variability can be attributed to several factors, including differences in task difficulty, environmental distractions, individual baseline attention skills, and the expertise of the assessor, as well as the subjective nature of the assessment task. Moreover, complex tasks may require advanced cognitive and motor skills that can be challenging for individuals with developmental disorders like autism (Varghese et al., 2023). In light of these challenges, researchers should explore the potential of simplifying JA assessment tasks while ensuring they remain ecologically valid. Simple tasks that closely mimic real-world environments have been found to produce more reliable and transferable results (Coleman et al., 2019). These tasks often involve everyday activities and interactions that are more familiar and natural for the individuals being assessed. For instance, VR environments can be used to create controlled yet realistic scenarios where participants can engage in JA activities without the extraneous variables present in a natural setting. Additionally, the use of technology such as eye-tracking and motion capture can enhance the precision of measurements, providing more consistent and objective data on JA performance. By reducing task complexity and leveraging advanced technological tools, clinicians and researchers can achieve a more accurate assessment of JA abilities, leading to better-targeted interventions and improved outcomes for children with ASD.
- *Longitudinal and developmental studies:* Longitudinal studies that track JA development over extended periods can offer valuable insights into its trajectory and the long-term effects of early interventions (Mundy et al., 1990). These studies are crucial for understanding how JA skills evolve in children with ASD compared to TD children. By following children over several years, researchers can identify critical periods for intervention, determine the stability of JA behaviors, and observe how early JA abilities influence later social and communicative outcomes. One key aspect of longitudinal studies is their ability to highlight the developmental trajectory of JA. Early identification of JA deficits can lead to timely interventions, which are known to significantly impact developmental outcomes. For example, research has shown that interventions focusing on improving JA in young children with ASD can lead to improvements in broader social communication skills and cognitive development (Kasari et al., 2010; Mundy & Jarrold, 2010). Identifying critical periods during which interventions are most effective can help clinicians tailor their approaches to maximize benefits during these windows of heightened neuroplasticity. Longitudinal data can also reveal how JA skills develop in different contexts and settings. For instance, the study by Mundy et al. (1990) found that children with ASD who received interventions aimed at enhancing JA showed significant improvements in their ability to engage in shared attention during play and learning activities. These findings suggest that JA interventions can have a broad impact on various aspects of social interaction, highlighting the importance of early and consistent support. Additionally, examining how early JA abilities predict later social and communicative outcomes can inform more effective and targeted therapeutic strategies. For instance, the study in Mundy et al. (2009) found that early JA skills are predictive of later language development and social competence in children with ASD. By understanding these predictive relationships, clinicians can design interventions that not only address immediate JA deficits but also support long-term developmental goals. For example, targeted JA interventions could be combined with language therapy to simultaneously promote verbal communication and social engagement. Moreover, longitudinal studies can help identify individual differences in JA development among children with ASD. This is important because ASD is a heterogeneous disorder, and children with ASD exhibit a wide range of abilities and challenges (Mundy, 2018). Understanding the factors that contribute to these individual differences, such as genetic, environmental, or cognitive influences, can lead to more personalized intervention strategies. For example, some children may benefit more from visual supports and structured activities, while others may respond better to play-based interventions that promote spontaneous social interactions. In addition to identifying critical periods for intervention and predicting long-term outcomes, longitudinal studies can also inform the development of new assessment tools and measures for JA. Traditional JA assessments often rely on cross-sectional data, which provide a snapshot of a child’s abilities at a single point in time. Longitudinal data, on the other hand, can capture the dynamic nature of JA development and reveal patterns of change over time. Future research in longitudinal studies of ASD will greatly benefit from leveraging technological advancements. The use of ML and DL can analyze large datasets collected over time, identifying trends and predicting developmental trajectories in JA, enhancing the prediction of developmental trajectories (Porayska-Pomsta et al.., 2018). Additionally, virtual and augmented reality (AR) technologies can create controlled, immersive environments for consistent behavior assessment (Jyoti & Lahiri, 2019). This can lead to the creation of more sensitive and comprehensive assessment tools that better reflect the complexity of JA behaviors in children with ASD.
- *Cross-cultural and diverse population studies:* Exploring JA in diverse cultural and socio-economic contexts is crucial for understanding its universal and culture-specific manifestations. Cultural contexts significantly influence how JA is initiated, maintained, and interpreted. For example, in some cultures, direct eye contact may be less frequent or may carry different meanings compared to Western norms (Grossmann, 2015). Recent literature indicates that social attention, particularly gaze cues, is influenced by participants’ cultural backgrounds. The amount and duration of eye contact can vary significantly across cultures in both everyday interactions and experimental contexts (Marchesi et al., 2023). For instance, East Asian individuals tend to engage in less mutual gaze compared to Western Caucasians during situations like business meetings or doctor–patient conversations, with averted gaze often seen as a sign of respect in East Asian cultures. However, studies by Haensel et al. (2020) have shown that contextual factors can alter these patterns, revealing that East Asians may engage in mutual gaze more than Western Caucasians in specific settings, such as storytelling tasks, and that gaze aversion can serve to reduce cognitive load during more demanding tasks. Moreover, many existing JA assessment tools are developed based on observations and norms from Western populations, particularly those from the United States and Western European countries (Marchesi et al., 2023). This raises concerns about their validity and reliability when applied across diverse cultural settings (Choi et al., 2016). Culturally specific norms and expectations are essential for accurately interpreting and assessing JA behaviors in non-Western contexts, as these norms significantly influence how children and caregivers engage in JA. In collectivist cultures, such as those found in parts of Asia and Latin America, communal goals often take precedence over individualistic pursuits, leading to implicit forms of JA that promote shared focus without explicit bids for attention (Greenfield, 2009). This contrasts with individualistic cultures like the United States, where autonomy and self-expression are emphasized, resulting in more direct JA behaviors (Greenfield, 2009). To address these challenges and ensure the validity of JA assessments across cultures, several strategies can be employed. 1) Incorporate culturally relevant behavior and norms into the current JA assessment tools. 2) Engage researchers from diverse cultural backgrounds in collaborative studies to develop frameworks for understanding JA across cultures (Bornstein, 2013). 3) Conduct longitudinal research to track the development of JA in children from different cultural backgrounds, taking into account environmental and familial influences (Carpenter et al., 1998).
- *Advanced technological approaches:* Advancements in technology, particularly in fields like computer vision, ML, and DL offer significant potential to enhance the precision, scalability, and ecological validity of JA assessments. Computer vision techniques can automatically analyze video recordings to detect and quantify JA behaviors such as gaze direction, gestures, and shared attention to objects or events (Zhang et al., 2018) ML algorithms trained on annotated datasets can accurately classify and track these behaviors over time, providing objective measures that are less reliant on human observation (Zhang et al., 2018). In addition, ML models can process large amounts of data quickly, enabling researchers to analyze JA in diverse populations across different contexts efficiently. This scalability is crucial for conducting studies with larger sample sizes or longitudinal designs, which are essential for understanding developmental trajectories and identifying atypical patterns early (Pusiol et al., 2014). Furthermore, DL models excel at learning complex patterns from data. By training on diverse datasets comprising various cultural and socio-economic backgrounds, these models can uncover universal and culture-specific patterns of JA development (Anzalone et al., 2014). This capability is particularly valuable for identifying early markers of JA deficits or deviations from typical development. Thus, AI-powered predictive analytics can assist in early diagnosis and personalized intervention planning for children with JA difficulties. By integrating data from multiple sources, including behavioral observations and physiological measurements, AI systems can provide assessments that account for individual variations and environmental factors (Varghese et al., 2024). For example, eye tracking can reveal gaze patterns to quantify JA, while heart rate variability (HRV) and electrodermal activity (EDA) can indicate emotional engagement and arousal levels during interactions (Billeci et al., 2018; Stuldreher et al., 2020). The utilization of VR and AR technologies offers controlled yet dynamic settings for assessing JA in more naturalistic contexts. These environments can simulate real-world scenarios where JA naturally occurs, such as play interactions or social gatherings (Coleman et al., 2019). This approach enhances the ecological validity of assessments compared to traditional laboratory settings. Moreover, VR and AR platforms can enhance engagement and interaction during JA assessments, particularly for children who may find traditional assessment methods intimidating or boring. By creating immersive experiences that mimic social interactions, these technologies can elicit more authentic JA behaviors and responses (Ravindran et al., 2019).
- *Personalized and adaptive interventions:* Developing personalized and adaptive interventions for JA based on individual assessment profiles represents a pivotal advancement in optimizing therapeutic outcomes for children. Tailoring interventions to the specific JA needs of each child is crucial because JA deficits can vary widely in presentation and underlying causes across individuals (Mundy, 2018). Detailed assessments, facilitated by advanced technologies such as ML and computer vision, can provide a clear understanding of an individual’s JA abilities, preferences, and challenges (Anagnostou et al., 2015). These assessments consist of various aspects of JA, including gaze following, pointing, and shared attention, which are essential for social communication and interaction. Personalized interventions leverage the insights from assessments to design targeted strategies that meet each child’s unique needs. For instance, children with ASD may benefit from interventions that incorporate visual supports, structured routines, and opportunities for practice in naturalistic settings (Kasari et al., 2010). Adaptive learning systems enhance this approach by dynamically adjusting the intervention content and pacing based on real-time feedback and progress monitoring (Essa et al., 2023). Such systems can modify the difficulty level of tasks, provide immediate reinforcement for correct responses, and adapt instructional strategies to optimize engagement and learning outcomes. By integrating personalized and adaptive interventions, practitioners can address individual variability in JA development effectively. This approach not only enhances the relevance and applicability of interventions but also promotes engagement and motivation among children undergoing therapy. Longitudinal studies underscore the importance of continuous assessment and adaptation throughout the intervention process to accommodate developmental changes and emerging needs (Schreibman et al., 2015). Furthermore, ongoing data collection and analysis enable clinicians to refine intervention strategies over time, ensuring sustained progress and positive outcomes in JA skills. In short, the future of JA interventions lies in personalized and adaptive approaches that capitalize on individual assessment profiles and leverage technology to deliver responsive and effective support. By providing interventions to address specific JA deficits and adjusting strategies in response to real-time feedback, practitioners can maximize therapeutic impact and foster meaningful improvements in social communication and interaction skills among children.
- *Ethical consideration and inclusion:* Addressing ethical and practical considerations in JA assessment is fundamental to developing fair and effective tools that can be widely applied across diverse populations. Accessibility is a key ethical consideration, ensuring that assessment methods are inclusive and accessible to children with varying abilities and cultural backgrounds. This involves designing assessments that accommodate different communication styles, sensory sensitivities, and developmental stages (Robins et al., 2001). Non-intrusiveness is another critical aspect, minimizing the burden on children and their families during assessments to avoid unnecessary stress or discomfort. Cultural sensitivity is paramount in JA assessment, as cultural norms and practices significantly influence social behaviors and interactions. Assessment tools should be adapted and validated across different cultural contexts to ensure they accurately capture JA behaviors in a culturally appropriate manner (Choi et al., 2016; Leung & Rheingold, 1981). Involving stakeholders such as parents, educators, and clinicians in the development process is essential. Their input helps ensure that assessments are not only scientifically valid but also user-friendly and relevant in clinical practice and educational settings (Wilkinson, 2016). Policy-level initiatives are also crucial for guiding ethical practices in JA assessment, particularly with the integration of AI and other advanced technologies. The FATE (Fairness, Accountability, Transparency, and Ethics) framework provides a robust foundation for ensuring ethical standards in ASD research and assessment (Kohli et al., 2022). Embracing stringent regulations and data protection measures helps mitigate risks such as data leakage and privacy violations, which are particularly pertinent when dealing with sensitive behavioral data from children. By fostering transparency and accountability in the development and use of JA assessment tools, researchers can build trust and confidence among stakeholders, promoting broader acceptance and adoption in clinical and educational settings.
- *Benchmark datasets and transfer learning:* The advancement of AI heavily relies on data, yet benchmark datasets remain scarce in autism research on JA assessment. Hence, establishing a collaborative research framework is crucial to improve existing datasets and develop new ones to share research outcomes globally. This initiative encourages collaborations among academics from various nations, fostering fair comparisons of technical solutions, joint experiments, and thorough empirical validation of studies on a worldwide scale. On the other hand, transfer learning involves leveraging knowledge learned from one task or dataset to improve performance on another related task or dataset, which is particularly valuable in JA assessment where data availability and variability are critical factors. For instance, the primary JA behaviors are gaze following, pointing, and showing (Mundy et al., 2017). Creating datasets specifically for these behaviors enables the extraction of features essential for training computer vision and DL models aimed at detecting these behaviors in children with ASD.

## Conclusion

JA plays a critical role in social communication and cognitive development, particularly during early childhood. It enhances language development by facilitating vocabulary acquisition, interpreting social cues, and developing conversational skills. Moreover, in educational settings, JA fosters engagement, collaborative learning, instructional effectiveness, and adaptive learning strategies. However, children with ASD often exhibit impairments in JA, which emphasizes the importance of JA assessment for early diagnosis and intervention. This review provides a thorough analysis of the methods used to assess JA, highlighting their strengths and limitations.

Initially, this review delves into methods used by clinicians and therapists to assess JA. These methods usually entail direct observation and structured tasks aimed at evaluating a child’s ability to initiate and respond to JA cues such as gaze following, pointing, and sharing attention. Subsequently, the review explores technological advancements that have revolutionized JA assessment. These innovative methods include eye-tracking technology, which tracks visual attention patterns during social interactions; VR, offering controlled yet realistic environments for assessing JA behaviors; robotics, utilizing interactive robots to engage children in JA tasks; and computer vision, employing AI algorithms to automatically analyze video recordings and detect JA behaviors.

While these approaches offer promising benefits such as enhanced objectivity, scalability, and ecological validity, they also present challenges. These include the need for specialized equipment, data privacy concerns, and the requirement for trained personnel to administer and interpret results accurately. Future research should focus on innovative technologies, validating their effectiveness across diverse populations and settings, and integrating them into clinical and educational practices seamlessly. It is important to note that this review focused extensively on discussing conventional and technological approaches to JA assessment, but did not explore into papers discussing intervention strategies, which were left as a topic for future exploration.

## Data Availability

The data supporting the review are available in the Open Science Framework (OSF) repository, https://osf.io/xjrbh.
